# Designing Anti-Influenza Aptamers: Novel Quantitative Structure Activity Relationship Approach Gives Insights into Aptamer – Virus Interaction

**DOI:** 10.1371/journal.pone.0097696

**Published:** 2014-05-20

**Authors:** Boaz Musafia, Rony Oren-Banaroya, Silvia Noiman

**Affiliations:** 1 Bio-Vent Ltd. Ashkelon, Israel; 2 Pontifax Inc, Herzelia, Israel; Ben-Gurion University, Israel

## Abstract

This study describes the development of aptamers as a therapy against influenza virus infection. Aptamers are oligonucleotides (like ssDNA or RNA) that are capable of binding to a variety of molecular targets with high affinity and specificity. We have studied the ssDNA aptamer BV02, which was designed to inhibit influenza infection by targeting the hemagglutinin viral protein, a protein that facilitates the first stage of the virus’ infection. While testing other aptamers and during lead optimization, we realized that the dominant characteristics that determine the aptamer’s binding to the influenza virus may not necessarily be sequence-specific, as with other known aptamers, but rather depend on general 2D structural motifs. We adopted QSAR (quantitative structure activity relationship) tool and developed computational algorithm that correlate six calculated structural and physicochemical properties to the aptamers’ binding affinity to the virus. The QSAR study provided us with a predictive tool of the binding potential of an aptamer to the influenza virus. The correlation between the calculated and actual binding was R^2^ = 0.702 for the training set, and R^2^ = 0.66 for the independent test set. Moreover, in the test set the model’s sensitivity was 89%, and the specificity was 87%, in selecting aptamers with enhanced viral binding. The most important properties that positively correlated with the aptamer’s binding were the aptamer length, 2D-loops and repeating sequences of C nucleotides. Based on the structure-activity study, we have managed to produce aptamers having viral affinity that was more than 20 times higher than that of the original BV02 aptamer. Further testing of influenza infection in cell culture and animal models yielded aptamers with 10 to 15 times greater anti-viral activity than the BV02 aptamer. Our insights concerning the mechanism of action and the structural and physicochemical properties that govern the interaction with the influenza virus are discussed.

## Introduction

In the drug development race against new influenza strains and emerging resistance to current drugs, there is a need for new drugs that act via new mechanisms of action on currently unexploited viral targets [1], [2], [3], [4], [5], [6]. Such a novel target is the hemagglutinin viral protein that facilitates the first stage of the influenza virus infection – the binding to a species-specific host cells in the respiratory epithelium. Recent reports have been published on the development of recombinant antibodies against hemagglutinin [7], [8], [9], but there is no small or synthetic molecule based drug that binds to and inhibits this target. A different approach employs Fludase, a recombinant chimerical enzyme that cleaves the sialic acid groups from the surface of the host’s respiratory epithelial cells: following that, the virus cannot attach to the host cell and the infection is inhibited [10], [11].

Jeon et al. [12] described an approach that focuses on the direct interaction and inhibition of the first stage of the influenza virus’ binding by blocking the viral hemagglutinin with synthetic DNA aptamers. They showed that aptamers that were selected against a specific peptide fragment of hemagglutinin were capable of inhibiting the hemagglutinin binding capacity of the virus, as well as preventing viral infection in tissue culture. Furthermore, these aptamers inhibited viral infection in an animal model. Employing this technology, BioVent has developed a treatment for influenza infection, using the aptamer BV02. In this work we used BV02 aptamer (named A22 in Jeon et al.) as the benchmark and starting point for the lead optimization process and the structure–activity study described in this publication.

Aptamers are single-stranded (ss) nucleic acid oligonucleotides (like ssDNA or RNA) that can form stable three-dimensional structures capable of binding to a variety of molecular targets with high affinity and specificity [13]. Aptamers are engineered through repeated rounds of in-vitro selection based on the binding to a molecular target such as a small molecule or a protein. The process of enrichment is called SELEX (systematic evolution of ligands by exponential enrichment) [14]. In a typical SELEX process, a random-sequence oligonucleotide library of 20–100 nucleotides long is synthesized. The library normally contains between 1×10^13^ and 1×10^15^ different oligonucleotide sequences. Only a few oligonucleotides out of this enormous pool strongly interact with the specific target and are therefore selected for enrichment [15].

In principle, aptamers may incorporate the advantages of biological drugs, such as single chain monoclonal antibodies, and of small molecule drugs. On the one hand, aptamers show the affinity and specificity of monoclonal antibodies, while presenting the chemical versatility of synthetic drugs, which may be modified using medicinal chemistry and also produced on a large scale synthesis.

To date, one aptamer has reached the market: Macugen, an anti-angiogenic drug for the treatment of neovascular age-related macular degeneration (AMD). Other aptamers are currently in clinical development [13], and many groups are developing anti-viral aptamers [16].

A typical aptamer is 5–15 kDa in size (15–45 nucleotides), binds its target with high affinity and can discriminate among closely related targets. Structural studies have shown that aptamers are capable of using the same types of binding interactions (e.g., hydrogen bonding, electrostatic complementarities, hydrophobic contacts, steric impediments) that drive the affinity and specificity in antibody-antigen complexes. Despite the fact that oligonucleotides are formed by only four nucleotides, it has been proven that this is sufficient to obtain a variety of three-dimensional structures and to achieve chemical versatility comparable to that of proteins, forming specific binding with virtually any chemical compound [17].

In general, it is necessary to have a 20% alteration of the original oligonucleotide sequence to switch any sequence from one secondary structure to another [18]. Aptamers are known to be very sensitive to changes in sequence; minimal sequence alterations (or mutations) may harm both the affinity of an aptamer to its target, especially if the mutations are in the regions that are considered to be the binding site of the aptamer to its target molecule [19], [20], [21], [22], or affect the secondary structure [20], [23].

An oligonucleotide’s secondary structure (2D) is mainly governed by its intra-molecular Watson–Crick base-pairing interactions [24]. The base pairing can be calculated and the secondary structure of the most stable structures of the oligonucleotide can be predicted. The oligonucleotides can adapt common structural motifs, which together compose the final structure, e.g. a) the stem & loop structure is comprised of complementary sequence regions of the same strand that form a well defined double helix that ends in an unpaired loop, b) external loops are series of unpaired bases without closing base pairs that therefore have no conformal constraints; they may be located between stems or at the edges of the oligonucleotide. Internal and external loops, and especially long loops, have less defined structures. Programs for 2D structure prediction calculate the best structure(s) based on thermodynamic considerations. The calculated ΔG value is one of the most used criteria for an *in silico* selection of 2D structures for a specific nucleotide sequence since the early works in the 1970s [25]. There are quite a few computer programs available for oligonucleotide 2D structure prediction especially for RNA. Mfold [26], [27], [28] and RNAfold [29] are the most relevant applications that predict the secondary structure of single stranded DNA or RNA.

In the present study we investigated the anti-influenza DNA aptamer BV02 and compared it to random (scrambled) sequence aptamers with similar length and other physical properties as controls. The scrambled aptamers were as active as the original BV02 aptamer and had similar binding affinity to the influenza virus. On the other hand, knocking down the 2D structure of BV02 diminished the affinity to the influenza virus, whereas the introduction of new structural elements increased the binding. These results raised the question whether the dominant characteristics determining the binding to the virus may not necessarily be sequence-specific as with other known aptamers [20]. This study explores the use of quantitative structure activity relationship (QSAR) with ssDNA aptamers for the first time. Furthermore, the study provides insights that may be relevant to development of anti-viral agents.

## Materials and Methods

### Oligonucleotides

DNA aptamers in small scale amounts were synthesized and purified by Ella-Biotech GmbH Germany. Medium scale amount for in-vitro studies was synthesized and purified by BioSpring GmbH Germany.

### Cell Culture and Viral Strains

Madin-Darby canine kidney (MDCK) (CCL-34, ATCC) were seeded into 6 well plates 10^6^ cells/well in growth medium [Dulbecco’s Modified Eagles Medium (DMEM) supplemented with 10% Fetal Calf Serum, streptomycin 0.1 mg/ml, penicillin 100 U/ml, mycostatin 6.25 U/ml, 0.06% glutamine] and incubated at 35°C 5%CO_2_, until cells were 80–90% confluent. The cells were washed 3 times in phosphate-buffered saline (PBS), and the medium was replaced with maintenance medium [DMEM, 2% heated inactivated FCS, strep 2000 u/ml, pen 0.1 mg/ml, mycostatin 6.25 U/ml, 0.06% glutamine and 0.0005% trypsin type IX]. Influenza virus stocks were added (50 to 100 µl/well) and incubated for 6 to 7 days at 35°C. After observation of cytopathogenic effect (CPE), the supernatants from infected wells were collected into one pool, divided to aliquots of 0.5 to 1 ml and store at −80°C.

The influenza strains used in this study were A/H3N2/Texas/1977; A/PERTH/265/2009 wild-type virus (H1N1pdm09; A/California/7/2009-like) and B/PERTH/211/2001 wild-type virus (B/Sichuan/379/99-like), the last two strains were kindly provided by isirv-Antiviral Group.

### Binding to Influenza Virus; Competition Assay between BV02 and Other Aptamers

BV02 binding assay to influenza virus bound to other aptamers (competitive assay): Biotinylated BV02 (5 nM, 100 µl/well) diluted in PBS (phosphate buffered Saline) was bound to Reacti-Bind NeutrAvidin coated 96-well (Pierce) for 30 min at room temperature (RT) in a rotor shaker. In parallel, tested aptamers were diluted in serial dilutions (to final concentrations of 0.5 to 500 nM) and incubated with the influenza virus (1∶1) for 30 min at RT. The biotinylated BV02 was washed 3 times in PBS with 0.05% tween-20 (PBS-T), and the aptamers/influenza suspension was added (100 µl/well) and incubated for 30 min at RT. The wells were washed 3 times in PBS-T, incubated for 1 hour at RT with Anti-FluA antibodies in blocking buffer, washed 3 times in PBS-T and incubated for 1 hour at RT with goat anti-mouse in blocking buffer followed by 5 final washes in PBS-T. Solution of TMB ELISA substrate (100 µl/well) was added for 20 min at RT and absorbance (OD) was measured at 652 nm.

### Inhibition of Influenza Virus Infection by Aptamers

MDCK cells were seeded in 96-well plates (2.5×10^5^/well) in maintenance medium and incubated for 2 to 4 hours at 35°C. The serially diluted aptamers and virus were pre-incubated together (1∶1) for 30 min at 35°C. After pre-incubation, the aptamers and influenza virus suspensions were added (50 µl/well) to MDCK cells and incubated for 3 days at 35°C. The cells were washed 3 times in PBS-T, incubated for 1 hour at RT with Anti-FluA (Anti-NP mouse monoclonal antibody; MAB8257 MsX Influenza A - CHEMICON) in blocking buffer (PBS-T with 1% Bovine serum albumin), washed 3 times in PBS-T, incubated for 1 hour at RT with goat anti-mouse (IgG conjugate to HRP; 115-035-071– JACKSON IMMUNORESEARCH) in blocking buffer, and finally washed 5 times with PBS-T. Solution of TMB (100 µl/well) was added for 20 min at RT and absorbance (OD) was measured at 652 nm.

### Inhibition of Influenza Viral Attachment to MDCK Cells in Cold

The method is similar to “Inhibition of influenza virus infection by aptamers” above, except for a modified incubation, which lasted 1 hour at 4°C.

The viruses were introduced into the host cells in low temperature, which enabled the first stage of attachment but not the second stage of fusion; in this way the amount of the viral particles on the cells’ surface corresponded only to the number of viruses that were attached to the cells, eliminating the effect of viral infection and replication. Moreover, the low temperature reduced the viral neuraminidase activity, which can release the virus particles from the cell surface, and minimized cell phagocytosis and pinocytosis that could have cleared the virus from the surface.

### Anti-influenza Efficacy of Aptamer in-vivo BALB/c Mouse Model

Aptamers were tested for their efficacy in an influenza infection model in BALB/c female mice. For testing the full effect of the aptamer and bypassing formulation issues, the aptamers were premixed with the virus and were compared to zanamivir as a positive control. Body weight loss served as the main parameter of disease severity [30]. All efforts were made to minimize animal suffering. The study was performed in the facilities of Pharmaseed Ltd, Ness-Ziona, Israel, after approval by “The Israel Board for Animal Experiments”, Ethics Approval Number IL-12-05-101, and in compliance with “The Israel Animal Welfare Act,”;.

Group size for the treated mice groups was n = 8, and typical body weight at study onset was 16–18 gr. Animals were acclimatized for 4 days and held at biosafety level 2 (BL2) unit in safety cabinets. The influenza A/H3N2/Texas/1977 inoculum, without aptamer, was calibrated to generate 10% body weight reduction by day 5. During the study, animals were observed daily for signs of morbidity and mortality. Body weights were recorded prior to dosing and at each day of the experiment.

Animals were sedated before treatment by ketamine-xylazine 85∶3 mg/kg mixture diluted 1∶3 in saline at a volume-dose of 0.1 ml/26 g body weight. Mice were inspected daily; as the regulations state, any animal found in a moribund condition or showing severe pain or enduring signs of severe distress (such as dyspnea, lateral recumbency, convulsions, plegia or inability to reach food or water) was humanely euthanized using carbon-dioxide asphyxiation. In addition, animals losing ≥20% of their initial body weight were also humanely euthanized. In case pain was suspected mice were injected subcutaneously with the opioid buprenorphine (0.1 mg/kg) or the non-steroidal anti-inflammatory drug carprofen (5 mg/kg), according to Pharmaseed SPOs.

BV02, BV35r, BV42 and zanamivir (Relenza GSK) were dilute in PBS to achieve 12.5, 9.8. 9.8, and 0.1 mg/Kg respectively. Each animal was administered 15 µl of virus mixture and 45 µl of treatment mixtures, or controls, that had been incubated together for 30 minutes at room temperature. The total volume of 60 µl per mice was administrated intranasally to the sedated mice.

### Structure and Thermodynamic Calculation with Mfold Program

The 2D structure calculation was done with the Mfold web version of Prof. Zuker [26]. http://mfold.rna.albany.edu.

The calculated free energies in Mfold are based on SantaLucia [31], the salt correction in Mfold is based on Peyret [32].

Standard protocol of linear ssDNA folding was used, at 35°C, with ionic conditions: [Na+] = 0.155 M and [Mg++] = 0.0 M. *(Note that the experimental binding assays were conduct at room temperature).*

### Training and Test Data Sets for QSAR Study

The training and testing sets were selected by sorting the database according to the experimental relative binding, then arbitrarily allocating every forth aptamer to the testing set, with the remaining aptamers allocated to the training set, as previously recommended [33].

In this way four options of training and test sets can be generated depending on the selection’s starting point. All the options gave similar results in the multiple-linear-regression model with regard to the type of descriptors and the quality of the model; here we present one of the training/test set options, in the supplement’s Table S1 the set of each aptamer is indicated. The full information can be found in Supporting Information file Data S1.

### QSAR Model

In order to find the mathematical relationship between aptamers’ structure and activity in the training set, two functions of MASS packages of R program were used (R 2.14.1 2011 The R Foundation). Firstly, a stepwise-selection with the function “stepAIC” (direction = both) focused on the most relevant 27 descriptors out of the 60 that can contribute to the prediction of the relative activity. Then, a second step with multiple-linear-regression function “lm” was applied on the selected descriptors to generate appropriate coefficients for the linear correlation equation. The quality of the output was evaluated according to the p-value of the null hypothesis, and multiple R^2^ indicated the correlation between actual and predicted values.

### Decision Trees Algorithm

In order to find a simple way to select aptemers with high affinity and avoid low affinity aptamers, a logical approach of Decision Trees was applied. Aptamers were considered as “high affinity” if their binding was about an order of magnitude better then aptamer BV02 (9 times greater or higher). Low affinity aptamers were considered molecules whose binding was twice that of BV02 or weaker, and medium affinity aptamers were between these two values.

The function “rpart” of the RPART package of R program (R 2.14.1 2011 The R Foundation) was used to select the relevant aptamer descriptors and the splitting-criteria appropriate for differentiation between the aptamers according to their binding range in a decision tree.

## Results

The relationship between the aptamer structure and its affinity to influenza virus was studied. For that, we have tested the influence of disrupting, or introducing specific 2D structures into, the aptamers by altering their sequence. This straightforward approach is described in the section SAR (structure activity relationship) bellow.

As more aptamers were synthesized and tested, enough evidence was gathered to allow the more quantitative approach: Quantitative Structure Activity Relationship (QSAR). At first, simple decision trees were used in designing the next aptamers for synthesis. Gradually, the gained information enabled the development of a full predictive model. This model also provided insights about the important structural elements that govern the binding of the aptamer to the virus.

QSAR is a tool commonly used in the optimization of small molecule drugs [34]. In general terms, one tries to find mathematical correlation between the biological activity and a series of properties of the molecule called descriptors. Descriptors, either calculated by computer models or measured, describe the structural and physical-chemical nature of molecules. The goal is to find a set of specific descriptors and a general functional equation that can be used to predict the molecular activity.

In an attempt to adapt the QSAR method to the aptamer world, the structural features of the aptamers that may affect the binding to influenza viruses were mathematically formulated. Algorithms that correlate the calculated properties of the aptamers with their activity were developed as described in the following QSAR section.

### SAR (Structure Activity Relationship)

Table 1 presents a selected list of aptamers and their relative binding. The table is organized in sets of related aptamers that elucidate the effect of changes of the tested structure on binding. Simple sequence–activity analysis reveled information regarding to 2D structures that enhanced or reduced the binding. About a hundred aptamers were synthesized and measured for binding to influenza virus (A/H3N2/Texas/1977). The full list of results and the sequences of the aptamers are shown in supplement Table S1.

**Table 1:** The contribution of structural elements of aptamers to their affinity towards influenza virus - based on SAR comparison between aptamers.

Set	Aptamer	Relativebinding	Length(bases)	
				**The insignificance of specific sequence (scrambled aptamers)**
	BV02	1.0	68	AATTAACCCTCACTAAAGGGCTGAGTCTCAAAACCGCAATACACTGGTTGTATGGTCGAATAAGTTAA
A	S1	1.1	68	GAAATAACCTCTGATAAAAAATTTCTCCATAGCGAAGTAAGTCCATAAGGAATGCCGATTCGGCTTGC
	S2	1.0	68	AGATAAATGTCCGGGTTCGCTTCACTGATCAAACATAGACAGGTATAATTACTGCCTGAAGAAAATCC
	S3	0.7	68	GTAAATACCTCTGAAATAATATCTCCATGACAGAGTAAGTCCATGAGAACTGACGATGCAAGTCTGTC
			68	**Importance of the size of the aptamer**
	BV02	1.0	68	AATTAACCCTCACTAAAGGGCTGAGTCTCAAAACCGCAATACACTGGTTGTATGGTCGAATAAGTTAA
B	BV19	1.3	68	AATTAACCCTCACTAAAGGGCTGAGT CTCAAAACCGCAATACACTCAGCCCTTTAGTGAGGGTTAATT
	BV19g	0.13	53	CTCACTAAAGGGCTGAGT CTCAAAACCGCAATACACTCAGCCCTTTAGTGAG
	BV19h	0.02	36	AACCCTCACT CTCAAAACCGCAATACAGTGAGGGTT
				**Loops of the 2D structure**
	BV02	1.0	68	ATTAACCCTCACTAAAGGGCTGAGTCTCAAAACCGCAATACACTGGTTGTATGGTCGAATAAGTTAA
C	BV14	1.3	68	ATTAACCCTCACTAAAGGGCTGAGTCTCAAAACCGCAATACACTGGTTGTACAACCAGTGTATTGCG
	BV13	0.08	68	TTTTGAGACTCAGC CCGGGCTGAGTCTCAAAACCGCAATACACTGGTTGTACAACCAGTGTATTGCG
	BV11	0.12	68	ATTAACCCTCACTAAAGGGCTGAGTCTCAAA ACGTTTTGAGACTCAGCCCTTTAGTGAGGGTTAATT
				**Repeated C nucleotide**
	BV24	15.0	30	CCCCCCCCCCCCCCCCCCCCCCCCCCCCCC
D	BV24a	0.08	30	CTCCTCCTCCTCCTCCTCCTCCTCCTCCTC
	BV24b	0.05	30	CACCACCACCACCACCACCACCACCACCAC
	BV24c	4.9	55	CCCCCCCCCCCCCCCCCCCCCCCCCCCCCCCCCCCCCCCCCCCCCCCCCCCCCCC
	BV24d	0.11	55	GGGGGGGGGGGGGGGGGGGGGGGGGGGGGGGGGGGGGGGGGGGGGGGGGGGGGGG
	BV24e	11.6	55	GGGGGG CCCCCCCCCCCCCCCCCCCCCCCCCCCCCCCCCCCCCCCCCCCCCCCCC
				**C-stretch perturbation**
	BV035a	14.6	55	AACGCTCACT CCCCCCCCCCCCCCCCCCCCCCCCCCCCCCCCCCCAGTGAGCGTT
	BV035m	6.5	55	AACGCTCACT CCCCCACCCCCCCACCCCCCCACCCCCCCACCCCCAGTGAGCGTT
E	BV035n	7.2	55	AACGCTCACT CCCCCCCCACCCCCCCCACCCCCCCCACCCCCCCCAGTGAGCGTT
	BV035o	9.9	55	AACGCTCACT CCCCCCCCCCCACCCCCCCCCCCACCCCCCCCCCCAGTGAGCGTT
	BV035p	9.9	55	AACGCTCACT CCCCCCCCCCCCCCCCCACCCCCCCCCCCCCCCCCAGTGAGCGTT
	BV035q	14.1	55	AACGCTCACT CCCCCCCACCCCCCCCCCCCCCCCCCCCCCCCCCCAGTGAGCGTT
				**C-stretch size**
	BV035h	10.4	59	AACGCTCACT CCCCCCCCCCCCCCCCCCCCCCCCCCCCCCCCCCCCCCCAGTGAGCGTT
	BV035a	14.6	55	AACGCTCACT CCCCCCCCCCCCCCCCCCCCCCCCCCCCCCCCCCCAGTGAGCGTT
F	BV035e	0.9	47	AACGCTCACT CCCCCCCCCCCCCCCCCCCCCCCCCCCAGTGAGCGTT
	BV035f	3.3	43	AACGCTCACT CCCCCCCCCCCCCCCCCCCCCCCAGTGAGCGTT
	BV035g	0.6	39	AACGCTCACT CCCCCCCCCCCCCCCCCCCAGTGAGCGTT
				**C-stretch location**
	BV35	1.01	55	CTCACTAAAGCGCTGAGT CCCCCCCCCCCCCCCCCCCACTCAGCGCTTTAGTGAG
G	BV35a	14.6	55	AACGCTCACT CCCCCCCCCCCCCCCCCCCCCCCCCCCCCCCCCCCAGTGAGCGTT
	BV35b	0.9	55	CCCCCCCCCGCGCTGAGTCCCCCCCCCCCCCCCCCCCACTCAGCGCGCCCCCCCC
	BV35c	1.2	55	CCCCCCCCCCCCCCCCCGCGCTGAGTCCCCCCCCCCCCCCCCCCCACTCAGCGCG
				**Test case: improvement of foreign aptamer**
H	C7-35M	0.5	35	GGTAGTTATAGTATATGGAAGGGGGTGTCGTATGG
	C7C	15.5	51	GGTAGTTATAGTATATGGAAGGGGCCCCCCCCCCCCCCCCGTGTCGTATGG

Relative binding refers to the ratio between IC50 of the tested aptamer and BV02 that served as a standard reference.

The relative binding is presented as the ratio between the IC_50_ values of the tested aptamer and parent aptamer BV02 (IC_50_[BVXX]/IC_50_[BV02]). The average IC50 of BV02 was 55 nM.

**Figure 1:**
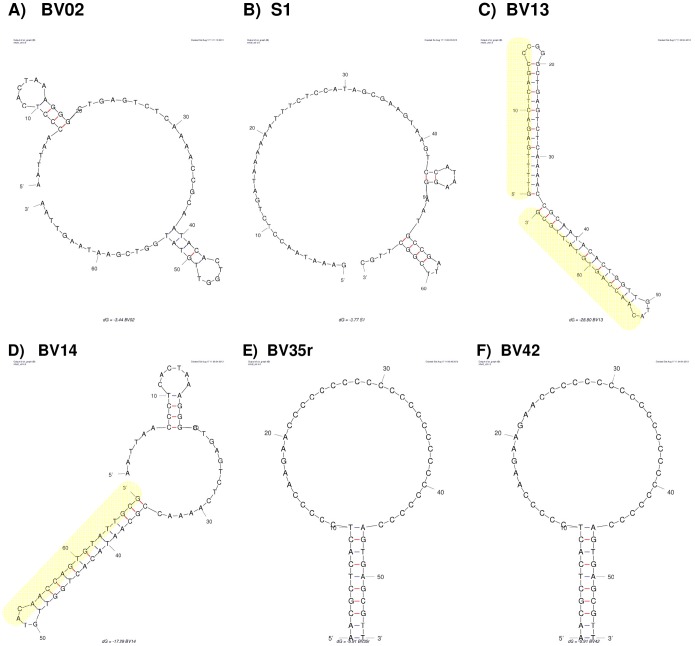
Secondary structure (2D) of aptamers calculated by Mfold. A) BV02 is the parent aptamer that was designed to influenza virus infection [12] by attaching the viral hemagglutinin protein. BV02 was selected based on its high affinity to a peptide segment of the protein. B) S1 aptamer is a scrambled aptamer with nucleotide ratio and chemo-physical properties similar to BV02 but with entirely different sequence. The binding of the random sequence aptamer to influenza virus was unexpectedly similar to BV02. C–D) BV13 and BV14 are based on BV02 sequence but the sequences that are marked in yellow were change to complementary sequence making new hairpin like secondary structures. BV13 have no internal or external loops and subsequently lost its affinity to influenza virus. BV14 maintained some of the 2D structure of BV02 and did maintain the affinity to the virus. E–F) BV35r and BV42 have large unpaired loops reach in C nucleotides. The binding affinity of these aptamers was about 15 higher than BV02. BV35r and BV42 were chosen for further investigation in-vitro and in-vivo and showed anti-influenza activity.

Set-A in table 1 shows the comparison of BV02 with **scrambled aptamers** that have entirely different sequence but with similar physical properties like size, nucleotide ratio and Tm (see figure 1-A and 1-B that show calculated 2D structure). Aptamers S1, S2 and S3 had relative binding similar to BV02 (1.1, 1.0, 0.7 respectively). The comparison indicates that the binding is not depended on the specific sequence of BV02. The scrambled aptamers S1–3 were not included in the following QSAR study.

Set-B in table 1 shows the **importance of the size** of the aptamer to the activity: the longer the aptamer the better was the binding. BV19, 19 g and 19 h have different lengths (68, 52 and 36 bases, respectively), and the relative binding decreased as the aptamer became smaller (1.3,0.13, 0.016 respectively).

Set-C in table 1 shows the attempt to find whether the **secondary structure** of BV02 aptamer is important to its relative binding to influenza virus. Parts of BV02 were replaced with complementary sequence making a hairpin structure instead of the original 2D structure. Introducing a hairpin structure to half of the aptamer and leaving internal and external loops untouched did not affect the binding of BV14 (see figure 1-D). Making the entire aptamer fold in to two hairpins with minimal loops reduced the relative binding of BV13 to 0.08 (see figure 1-C). Folding the aptamer to a single long hairpin reduced the relative binding of BV11 to 0.12. These results indicate that the un-paired part of the aptamers; namely the internal and external loops of the 2D structure, are important for its affinity to influenza virus.

Set-D in table 1 shows the importance of **repeated C nucleotide** stretches to the relative binding. The binding of the pure 30 C-repeat of aptamer BV24 was high, and better than aptamers with repeated sequence that are interrupted with T (poly CTC), as with BV24a, or A’s (poly CAC), as with BV24b. (15.1, 0.08 and 0.05, respectively). The binding of the 30 repeats pure C BV24 aptamer was better than aptamer BV24c, which comprised 55 C-repeats (15.1, 4.9). The 55 G-repeat aptamer BV24d had low binding (0.11). Combination of 6 Gs and 49 Cs in BV24e gave new defined 2D structure and better binding (11.6). This may indicate that C-repeats, as long as these are not too long, contribute to the binding by an order of magnitude, through either sheer addition of length, like in the case of BV24, or by secondary structure as with BV24e. In opposite to pure C-repeats sequence, a worse binding was showed by repeated G, CTC or CAC sequences, indicating that G, T or A nucleotides cannot replace C nucleotide.

Set-E shows the effect of **perturbations of the C-stretch**. Single (BV35p) and double (BV35o) interruption of an adenosine instead of cytosine in the center of the C-stretch loop reduced the relative binding to 9.9 in both cases in comparison to non-interrupted (BV35a) sequence (14.6). The effect was more pronounced with the triple interruption of BV35n (7.2) and with four interruptions as in the case of BV35m (6.5).

The position of the perturbation is important as well. The perturbation of BV35q is located at the side of the loop and the relative binding was not affected (14.1), BV35p have perturbation at the center of the C-stretch loop and its relative binding was reduced to 9.9.

Set-F compares the effect of the **size of the C-stretch** loop and the total length of the aptamer with constant 10 base-pairs stems, (stems are underlined). BV35h is 59-base long with 39 C’s, and BV35a is 55-base long with 35 C’s; these long aptamers had higher relative binding (10.4 and 14.6, respectively) than BV35e, BV35f and BV35g that were, respectively, 47-, 43- and 39-base long with 27, 23, 19 C’s (0.9, 3.3 and 0.6, respectively). This suggests that longer aptamers with longer C-stretches have better binding. It seems that a C-stretch length between 55 and 47 bases denotes the threshold separating high affinity aptamers from low affinity aptamers. It should be noted that the longer BV35h did not show better binding than BV35a, therefore an optimum of length seems to exist. This is similar to the abovementioned case in which the pure C aptamer BV24, with 30 bases, showed better binding than the BV24c with 55 bases.

Set-G shows the importance of the **location of the C-stretches** in the secondary structure. BV35 has a C-stretch of 19 C’s; when the length of the C-rich loop was increased to 35 bases the relative binding increased from 1.01 to 14.9 (BV35a). In order to test whether the numbers of the C nucleotides and their position were important, we designed BV35b and BV35c that have total of 35 C’s, 16 in external loops, 19 in internal loops, with different paired sequence. The relative binding BV35b and BV35c were 0.94 and 1.16, respectively, similar to BV35 (1.01), which has only 19 C’s in a single loop. In this case, the external C-stretch did not contribute to the binding. Thus it may be important that the C-stretches are concentrated in loops or in one region as in BV24e.

Set-H presents a **test-case: Improvement of an aptamer described in the literature.** Introduction of 16 C’s into an aptamer taken from the literature is shown. The aptamer C7-35M was produced against hemagglutinin of the H9N2 type avian influenza [35], and had low relative binding of 0.5 in our system. After adding 16 Cs inside the aptamer sequence, the relative binding increased to 15.5 for the C-rich aptamer C7C. This indicates that the importance of C stretch can be generalized, and that it may be relevant to any aptamer.

### QSAR Models

The insights gained from the comparisons of aptamers and their binding to influenza viruses taught us that certain general structural motifs are governing the interaction rather specific sequences. To investigate the relative importance of each component that contributes the binding, a full mathematical model was needed. QSAR provided a mathematical method to examine whether the structure–activity relationship could be quantified and serve for predicting the binding affinity of aptamers to the influenza virus. The structural and physicochemical nature of the molecules were represented by selected descriptors (parameters). The structure and the thermodynamic properties of the aptamers were calculated using the program mFold as described in Materials and Methods section.

Each of the 60 structural descriptors was calculated as listed in the supplement’s Table S2. These descriptors can be classified as:

- General information that may be extracted without any structural calculations, like size, number of each nucleotide or chemical modifications.
- Thermodynamic descriptors that describe the energetic properties and melting temperature (Tm) of each conformation (note that in case there were several stable conformations for the same aptamer an average value was used; in case there was one dominant conformation that was more stable than the others the stable conformation was chosen).
- Descriptors that refer to the C-nucleotide stretch, like size, orientation, interruption of the stretches by other sequences and un-paired fractions.
- 2D structural descriptors, like the number, size and position of internal and external loops (e.g. un-paired regions) and stems (e.g. paired regions).
- Proportions (ratios) between the above descriptors and the size of the aptamer, or ratios between various descriptors.

For each of the first three stems and loops (ordered according to their size), the size of the loop and stem, the thermodynamic contribution to the conformation, and the perturbations as bulbs to the stem and loop were calculated.

In the preliminary analysis we found that thermodynamic variables like Tm, ΔG, ΔH, and ΔS, which were calculated by the 2D prediction program, were not predictive with regard to the affinity to the virus. Moreover, since aptamers comprised of poly-C, poly-G, poly-CTC and poly-CAC cannot form Watson & Crick base pairs, the programs could not produce 2D structure and calculate thermodynamic descriptors. For these reasons, we did not include the thermodynamic properties in the final process of QSAR development; the most relevant 27 descriptors were chosen to further selection.

The correlation function was developed based on a training set of 72 aptamers. The test set included 24 aptamers (see Materials and Methods).

After stepwise model selection, a multiple regression model was generated based on the training set. The model included nine descriptors, with a correlation of R^2^ = 0.7427, p-value: 3.531*10^−15^. We have found that deleting the descriptor that counts the number of T’s in the aptamer did not significantly weakened the correlation; this descriptor appears to have no clear relevance. The reduced model is presented in equation-1 (Eq1), which includes eight descriptors (see table 2), with R^2^ = 0.705, p-value: 4.5*10^−14^.
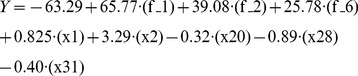
(1)


**Table 2:** Descriptors relevant to aptamer binding to influenza virus.

Name	Descriptor explanation	Descriptor Rangeof values			
		Average	Max	Min	SD
x1	Total number of Bases	55.41	69	30	9.16
x2	Number of calculated conformations	1.08	4	0	0.59
x15	Length of longest un-paired C-stretch that may be perturbed with anysequence that is no longer than 8 nucleotides	17.17	55	0	15.78
x20	Sum of un-paired nucleotides in C-stretches comprised of 3 Cs or more	17.04	55	0	16.10
x28	Sum of all un-paired nucleotides	33.24	55	4	9.03
x31	Size of largest loop	22.21	39	0	12.58
f_1	Ratio between Sum of all un-paired nucleotides - And -Total base number = x28/x1	0.613	1	0.059	0.170
f_2	Ratio between Size of largest loop - And -Total base number = x31/x1	0.409	0.778	0	0.240
f_6	Ratio between Length of longest un-paired C-stretch that may be perturbedwith any sequence that is no longer than 8 nucleotides - And - Total basenumber = x15/x1	0.325	1.000	0	0.301

The full list of the descriptors that was extracted from the calculation with Mfold and analysis of the sequence is presented at supplement Table S2.

**Figure 2:**
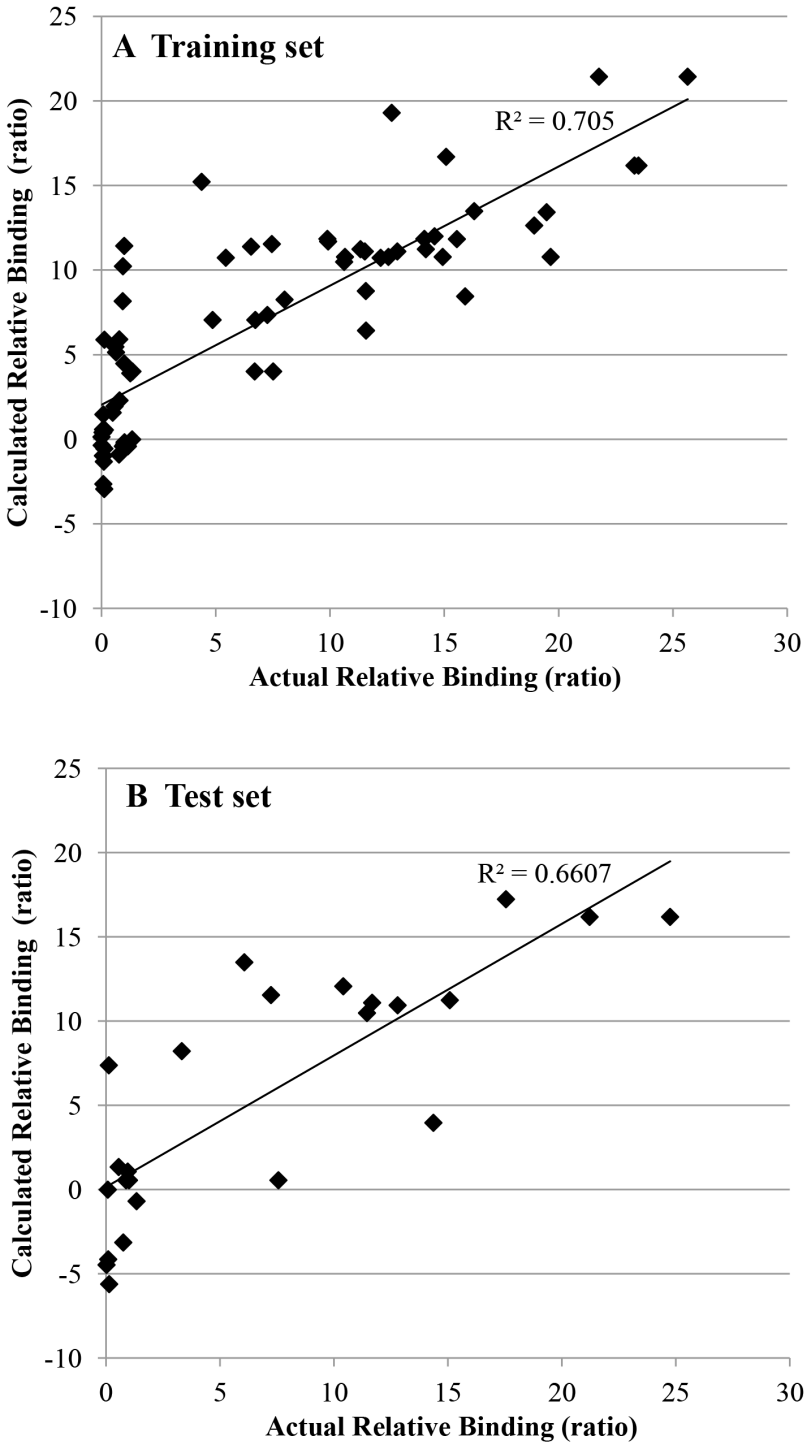
The actual versus predicted relative binding of aptamers in training and test sets. A) The training set actual vs. calculated chart can be seen, with R^2^ = 0.705 p-value: 4.5*10^−14^. The most important finding is the ability of the model to predict accurately which aptamers are likely to have high relative binding. The sensitivity value was 89% for the identify molecules with high relative binding (at least 9 better than BV02), and the specificity of the model was 87%. B) The model predicted well the test set as well, and showed sensitivity value of 89% for the identify molecules with high relative binding and the specificity of the model was 87%; the correlation was R^2^ = 0.66.

The plot of the actual versus predicted relative binding of the training set is presented in figure 2-A. As can be seen, the model managed to predict the activity of the aptamers. In most cases the model managed to separate between high- and low-binding molecules. The model showed very good sensitivity of 89% for the training and test sets, indicating the ability of the model to identify molecules with high relative binding (at least 9 aptamers were better than BV02). The specificity of the model was 87% for the training and test sets, indicating good ability to differentiate molecules that do not have high relative binding from those that do.

The model was applied to the test set and showed good correlation of R^2^ = 0.66. The plot of the actual versus predicted relative binding of the test set is presented in figure 2-B. Among the aptamers in the test set, 8 out of the 9 predicted to have high binding had indeed high binding; none of the aptamers with empirical low binding had been predicted to have high binding. This makes this model useful in designing new aptamers. The model provides an algorithm that predicts the affinity of aptamers to influenza virus and proves that quantitative structure-activity relationship study of ssDNA aptamers can be efficiently performed.

**Figure 3:**
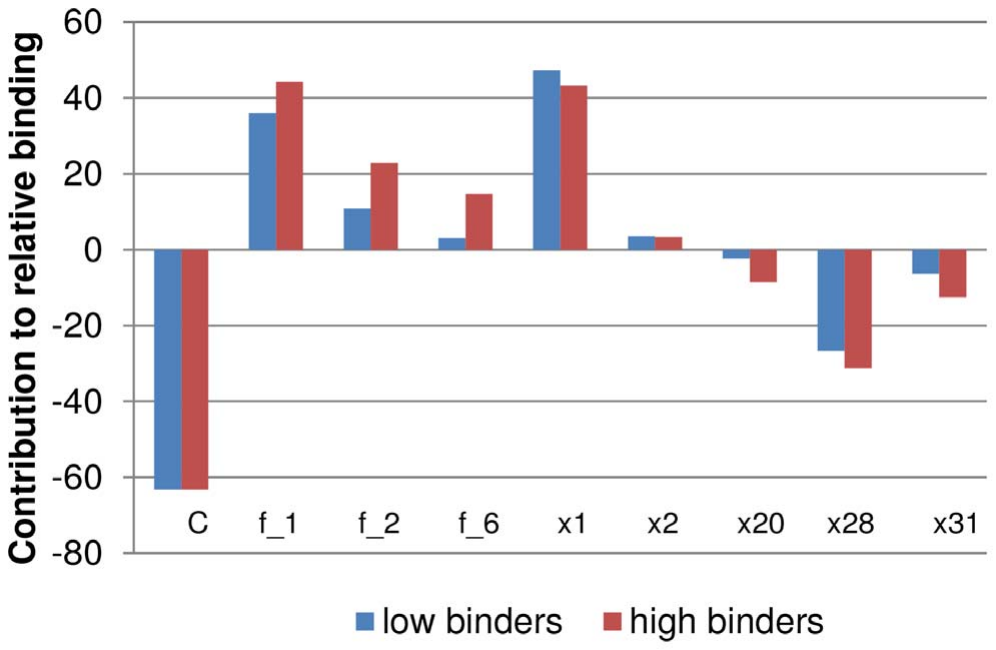
The contribution of each descriptor to the relative activity model. Equation 1 gives the prediction model of the calculated binding relative to BV02. Each descriptor after multiplying its value with the coefficient in the linear equation Eq1 has negative or positive value that contributes to the calculated prediction. In order to elucidate the structural features that improve the binding of aptamer to influenza virus, the chart above presents the average contributions of aptamers that had high affinity in comparison to aptamers with low affinity. (High affinity are aptamers that their relative binding was 9 times better or more then aptamer BV02; low affinity are aptamers with relative binding of 2 or less). The most profound difference that indicates the positive contribution structures to binding of the aptamers to influenza virus can be seen in f_2 and f_6 descriptors. These descriptors relates to the size of the largest loop and the size of unpaired C-stretches respectively.

The descriptors that were selected for the model are listed in table 2. The contributions of the descriptors are presented in figure 3. The histogram describes two groups: aptamers with high relative binding to influenza virus (at least 9 times the affinity of BV02), and aptamers with low or equivalent binding (no more than 2 times BV02’s affinity). Descriptors f_1, f_2, f_6, x1 and x2 positively contribute to the equation. Descriptors x20, x28 and x31 had negative coefficients; the constant C was negative as well. It is interesting that descriptor f_1, the ratio between all un-paired nucleotides and the aptamer length (x28/x1), was counterbalanced by x28, which itself had a negative coefficient. Similar relationship was seen with descriptor f_2, the ratio between the size of largest loop and the aptamer length (x31/x1), which was counterbalanced by x31 which had a negative coefficient. Descriptor f_6, the ratio between the length of longest un-paired C-stretch (with or without some perturbation) and the aptamer length (x15/x1), was counterbalanced by x20 that describes the total length of the un-paired C-stretches. Thus the model can be written as presented in Eq 2, using only six descriptors in a non-linear equation. Attempts to omit the ratio-based descriptors gave unsatisfactory models. This may indicate that the correlation between the descriptors and the binding is not simply linear.
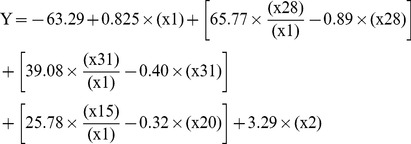
(2)


The equation indicates that the binding of an aptamer to influenza virus is related to the size of the aptamer, the amount of unpaired nucleotide, the size of the largest internal loop, the length of unpaired C-stretches and the number of stable conformations calculated.

**Figure 4:**
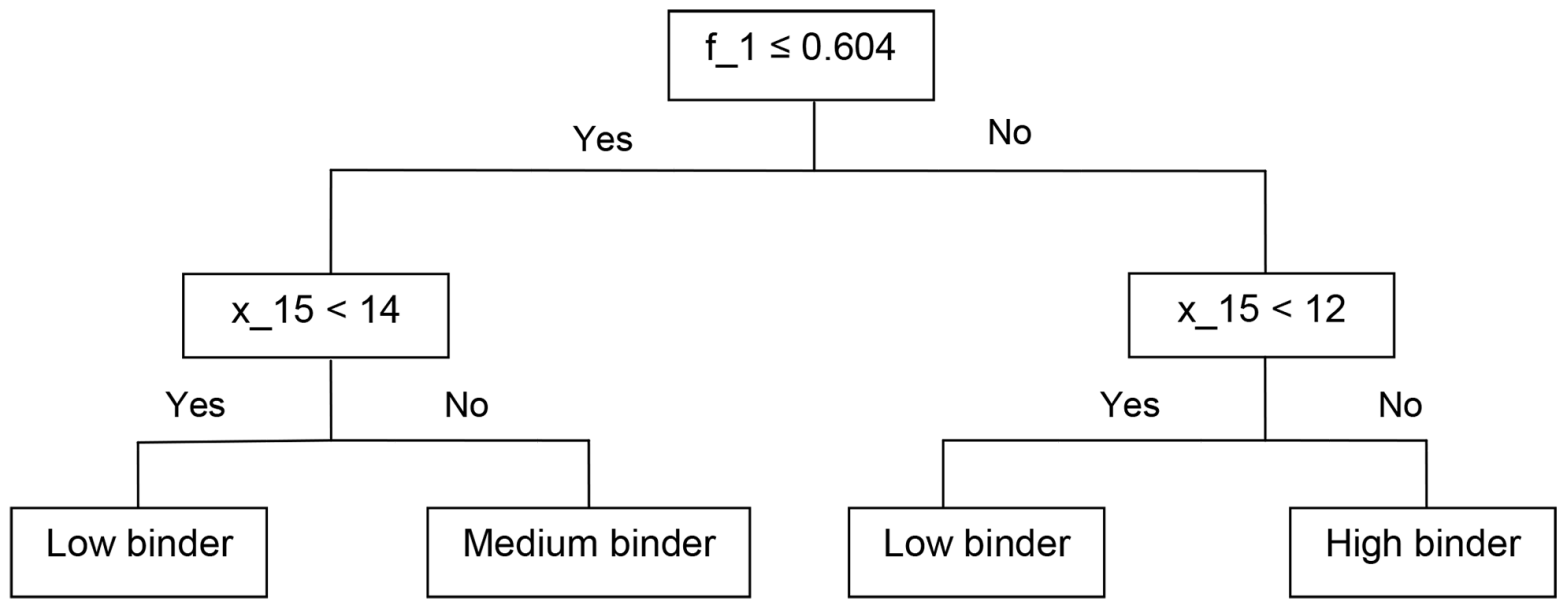
Decision tree for selecting aptamers that may have high binding affinity to influenza virus. Decision trees were used during the aptamer optimization process as a tool to select the aptamers that should be synthesized and examined. The decision trees helped to avoid aptamers with low affinity or to enrich the aptamers with high affinity. The recursive partitioning algorithm was used to select descriptors that better differentiate the aptamers according to their binding range (see materials and methods). This tree used only two descriptors f_1 and x15 and had high sensitivity, thus it has low probability to miss high affinity aptamers. The accuracy of this model is described in table 3.

During the process of lead optimization we have used decision tree method that gave rules of thumb for focusing on the more active aptamers. This tool was employed only on a single (training) set as it was used along the process of the optimization when only few dozens of molecules were available, and gave prediction whether the expected binding was low, medium or high. The decision tree method provided a logical and simple approach to select the higher affinity aptamers and to discard those with low affinity. In figure 4 an example of one of the Decision Trees used in the lead optimization process is presented: this tree had 95% sensitivity and 82% specificity to aptamers with high relative binding, e.g. it was unlikely to miss such an aptamer. Such a tree was found useful in selecting compounds, with low probability to miss the most active molecules.

An evaluation of the predictivity of the decision tree is presented in table 3; it shows the sum of correct or false predictions based on the comparison of the calculated prediction of each aptamer to its actual binding.

**Table 3:** Analysis of decision tree accuracy: predicted versus actual binding.

		Calculated Low(a)	Calculated Medium(b)	Calculated High(c)
**Actual Low** (a)	**(47)** (d)	36^(f)^	7	4
**Actual Medium** (b)	**(14)**	4	1	9
**Actual High** (c)	**(37)**	0	2	35(e)

(a) High affinity are aptamers that their relative binding was 9 times better or more then aptamer BV02 (>9).

(b) Medium affinity are aptamers that their binding was between low and high (2< binding ratio <9).

(c) Low affinity are aptamers that their binding was in the range of BV02 or worse than twice its binding (<2).

(d) The numbers in brackets indicate the number of the aptamers according to their experimental binding.

(e) The decision-tree predicted well (35/37) high affinity aptamers, (f) but was less successful in detecting the low affinity aptamers (36/47).

This tree used two descriptors f_1 (Ratio between Sum of all un-paired nucleotides And Total base number) and x15 (Length of longest un-paired C-stretch that may be perturbed with any sequence that is not longer than 8 nucleotides). The rule of thumb derived from the decision-tree says that if one does not want to miss aptamers with good binding, one should test those having unpaired region that is more than 60% of the sequence and C-stretch that is longer than 12 nucleotides.

### Lead Optimization

The SAR and QSAR studies were part of a lead optimization process that was aimed at producing new lead candidates for further development as anti-influenza agents; aptamers with at least one order of magnitude greater binding than BV02 to influenza virus were sought. We found 14 aptamers that had 15 times greater affinity than BV02, of which 6 had affinity that was more than 20 times greater. The selection of the candidates was based on their experimental binding affinity to influenza virus (in terms of IC_50_), activity against a spectrum of strains, bio-analytical method sensitivity (qPCR), safety and cost of production.

Two candidates were selected: BV35r and BV42, with IC_50_ of 5.0 nM (±1.2) and 4.0 nM (±0.4), respectively, which is, respectively, 11 and 14 times greater than BV02’s IC_50_ of 55.7 nM (±10.9). BV35r and BV42 are 55 nucleic bases long, in comparison to the 68 bases of BV02.

The sequence of BV35r is: AACGCTCACTCCCCCAAGAACCCCCCCCCCCCCCCCCCCCCCCCCAGTGAGCGTT.

The sequence of BV42 is: AACGCTCACTCCCCCAAGAAGAACCCCCCCCCCCCCCCCCCCCCCAGTGAGCGTT.

These aptamers were subjected to further studies.

### The Mode of Action of the Anti-viral Activity of the Aptamers

**Figure 5:**
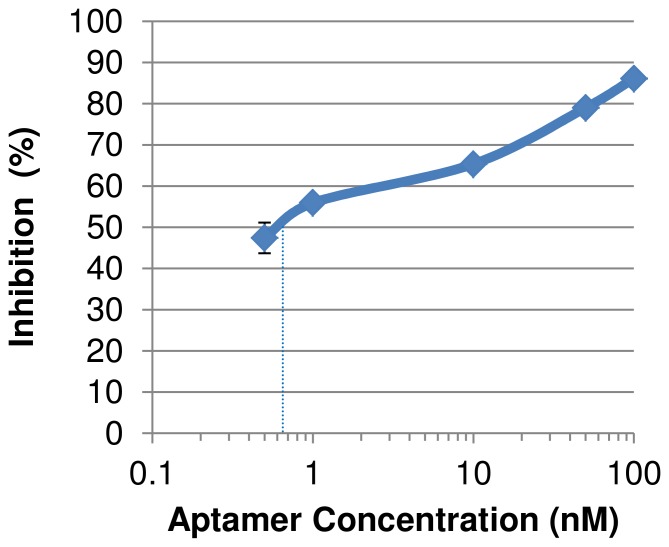
The inhibitory effect of aptamer BV42 on the first stage of viral attachment to MDCK cell. BV42 managed to inhibit the attachment of influenza virus (A/Texas/1/77) to MDCK host cells. The attachment of the virus to the host cells was reduced by BV42 aptamer in a dose dependent manner, the IC50 was about 1 nM. To show that the aptamer is affecting the first stage of the infection, the attachment of the virus to the host cells was done in 4°C; in this way the amount of viruses on cells refers to viruses that are attached to cells alone without the effect of the next stages of the viral infection cycle. The amount of the virus attached to the cell culture is expressed as optic density (OD) of the colorimetric product that is produced by enzyme attached to anti-influenza antibody. The inhibitory effect = 100 * [1–OD_(test)_/OD_(no aptamer)_] The error bar indicated standard error (SE).

The viruses were introduced into the host cells in low temperature, which enabled only the first stage of attachment. The attachment of the virus to the host MDCK cells was reduced by BV42 aptamer in a dose-dependent manner with an IC50 of ∼1 nM, as can be seen in figure 5. The data indicate that the aptamer acts through direct binding to hemagglutinin, which prevents the attachment of the virus to the cell. This paradigm was supported by qualitative experiment testing the direct binding of purified recombinant hemagglutinin H1 influenza protein to immobilized BV42 aptamer (data not shown).

### The in-vitro and in-vivo Biological Activity of the Aptamers

**Figure 6:**
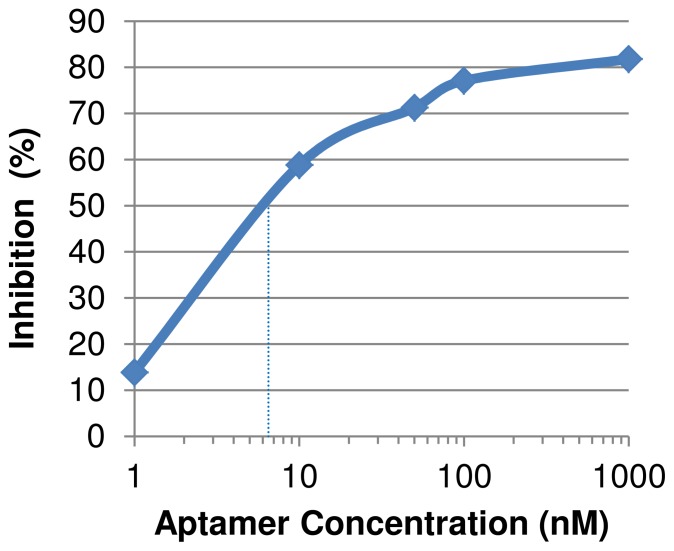
The inhibitory effect of aptamer BV42 on swain flu infection of MDCK cells. BV42 inhibited the infection of host cells by the 2009 pandemic swain flu virus (H1N1 A/Perth/265/09); with EC50 of about 8 nM. MDCK cells infection was measured according to levels of virus in the cell culture as described in figure 5. The error bar indicated standard error (SE).

It was found that aptamers interacting with hemagglutinin inhibit the infection of cultured cells by the virus. The inhibition of influenza (H1N1 A/Perth/265/09) infection was shown to be dose dependent with an EC50 of 8 nM. The levels of the viruses measured in infected MDCK cells are presented in figure 6. Aptamers BV35r and BV42 also inhibited the infection of influenza type B (B/PERTH/211/2001) with an EC50 of 5 nM and 30 nM, respectively (supplement’s Figure S1).

**Figure 7:**
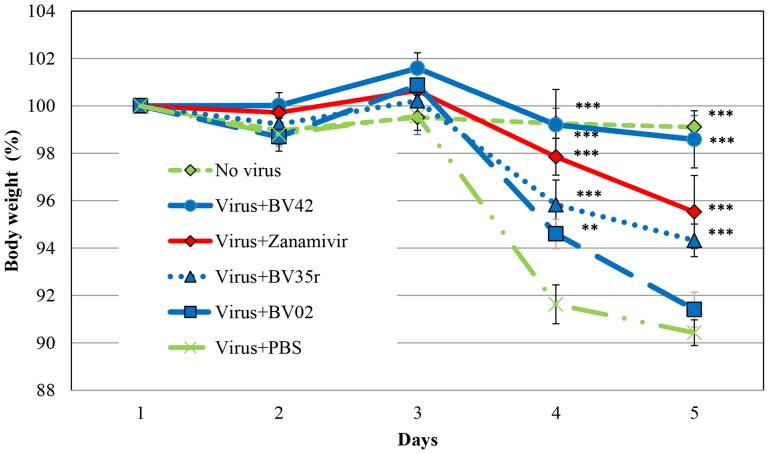
The anti-viral effect of aptamers in mouse model. The anti-viral effect of aptamers BV02, BV35r and BV42 and the anti-influenza drug zanamivir is presented. The severity of the infection was monitored according to the body weight change of each mouse along the experiment. Single intranasal treatment of equimolar amount of BV02, BV35r, BV42 (0.8 µmol/Kg) were administrated (12.5, 9.8. and 9.8 mg/Kg respectively) and 0.3 µmol/Kg of zanamivir (0.1 mg/Kg), PBS buffer served as placebo. The viral inoculum and treatment were co-administrated intranasally. BV42, BV35r and zanamivir showed significant improvement efficacy in comparison to mice treated with placebo on days 4–5. BV42 showed better effect in comparison to the other aptamers (p<0.001). The statistics is based on ANOVA for repeated measures and Post-hoc Bonferroni’s statistical analysis, (**p<0.01, ***p<0.001). The error bar indicated standard error (SE).

To further evaluate the inhibitory effect on influenza infection, the aptamers were tested in a mouse disease model. The results are presented in figure 7. This animal model focused on the antiviral activity of the aptamer following intranasal administration and bypassed bioavailability and formulation issues by mixing the aptamer and the virus before inoculating the animals intranasally. (Non-stabilized aptamers like the ones we used in this study have short half-life of <30 minutes when administrated intravenously, probably due to renal excretion). The loss of the mice bodyweight indicated the severity of the infection.

Figure 7 describes the effects of BV02, BV35r, BV42 and the commercially-available anti-influenza drug zanamivir (at 12.5, 9.8. 9.8, and 0.1 mg/Kg respectively, which is equivalent to 0.8 µmol/Kg for the aptamers and 0.3 µmol/Kg for zanamivir) on the mice body weight during experimental influenza infection. The mice were inoculated on day 1. The body weight change from day 1 (prior to inoculation) was monitored in each mouse for five days. BV42 and BV35r showed significantly improved efficacy compared to BV02. The effect of BV42 was dose dependent with maximal activity at 10 mg/Kg (supplement’s Figure S2).

The biological studies indicate that the QSAR methods produced novel aptamers with better binding to influenza; furthermore, the biochemical improvement was translated to enhanced biological activity of viral infection inhibition, in cell culture and animal models.

## Discussion

The aim of this work was to optimize the lead aptamer BV02 as a therapy against influenza virus infection. We have managed to produce aptamers that had 10 to 15 times greater influenza hemagglutinin binding affinity than BV02, as well as better anti-infective activity in biochemical, cell culture and animal influenza models. As this process advanced, the special nature of the aptamers’ binding to the viral hemagglutinin became clearer, and we were able to characterize this binding behavior in mathematical terms as a function of the aptamer’s structure, thus providing for the first time an example of employing QSAR study in the oligonucleic world, in addition to sequence alignment bioinformatics tools.

### QSAR

Sixty descriptors quantifying structural and thermodynamic properties of the aptamers were produced and gradually the most relevant descriptors were selected to form a six descriptor- based algorithm, which can predict the binding of aptamers to influenza virus and thus anticipate its anti-viral activity. The algorithm successfully predicted the binding affinity in the training set of aptamers (R^2^ = 0.702) and the was validated with a test set (R^2^ = 0.66); moreover, the model’s sensitivity and specificity in selecting aptamers with enhanced relative binding were high, 89% and 87%, respectively. To our knowledge it is the first time that a QSAR study based on 2D data was successfully employed on ssDNA aptamers.

In this article we also present one example from several simple decision trees that were developed and served during the lead-optimization study. The decision trees, based on two or three descriptors, predicted whether an aptamer is likely to have good binding affinity and thus appropriate for synthesis and testing.

Analysis of the descriptors that were selected by the QSAR process indicates that the most important descriptors are related to the size of the aptamer, the amount of un-paired nucleotides, the size of the largest internal loop in the 2D structure, the amount of un-paired C stretches and the numbers of the conformations generated in the 2D calculation.

The size of the better-binding aptamers was relatively large: while the normal size of aptamers is between 15 and 45 nucleotides, our aptamers were larger than 45 nucleotides. The amount of un-paired nucleotides in either external or internal loops strongly contributed to the aptamer activity: on average 61% of the nucleotides were un-paired in all the better-binding aptamers. The C stretches also contributed to the binding affinity, with a minimum of eight C nucleotides, but preferably with twenty or more such nucleotides (though these stretches can be moderately interrupted by other nucleotides). These quantitative rules may teach us about the factors governing the interaction between the aptamers and influenza virus. The QSAR approach presented in this study may be less relevant to standard aptamers that show sequence sensitivity; however, it may offer an alternative approach to non-standard cases and lead optimization.

### The Nature of the Aptamer and Influenza Virus Interaction

Unlike other known aptamers, our oligonucleotides’ binding to hemagglutinin was unexpectedly non-sequence-dependent but rather depended on certain non-specific secondary structures, such as loops and C stretches. Moreover it seems that there is no defined binding site on the aptamer. The aptamers inhibited the first stage of the virus – cell interaction, as indicated by the experiment in low temperature cell culture. This inhibition could have been the result of different mechanisms, including competitive and non-competitive inhibition of hemagglutinin, and interference with other parts of the virus. For this reason it is less likely that viral resistance due to escape mutants will occur, because the interaction is probably not limited to a single binding site that can be changed by a single point mutation.

Loops either external or internal have relatively loose structure since they comprise flexible un-paired ssDNA. The C stretches that contributed to the affinity of the aptamer were made of repetition of C nucleotides, which is the most compact and hydrophilic of all nucleobases, therefore it is expected to enable maximal flexibility to the oligonucleic backbone with minimal steric hindrance. All this may indicate that structural flexibility is an important factor in the binding of aptamers to the virus.

Random oligonucleotides (randomers), that also do not have a defined binding site, are known to have anti-viral effects. Vaillant et al. [36] have showed that phosphorothioate oligonucleotides inhibit hepatitis C (HCV) and HIV virus fusion with the host cells; they related this effect to the amphiphilic nature of the phosphorothioate oligonucleotides. In the current work, we also showed that adding phosphorothioate groups to BV02 moderately improved binding (aptamers BV29 and BV31 in the supplement’s Table S1).

Vaillant et al. also found that phosphorothioated DNA randomer is more potent than phosphorothioated 2′-O-methyl-RNA and 2′-O-methyl-RNA which are not active at all. [36], [37] Furthermore, Bernstein et al. [38], [39], showed the anti-viral activity of randomers and poly-C phosphorothioate DNA oligonucleotides against a wide variety of viruses. It should be noted that oligonucleotide have poly-anionic nature, and it is well known that poly-anionic polymers may have antiviral activity. [40], [41], [42], [43].

Unlike ssDNA aptamers, RNA based aptamers that were developed against hemagglutinin showed specificity and selectivity to particular influenza strains [44], [45]; thus the general anti-influenza activity described in this paper may be specific to DNA-based aptamers.

Contrary to random polymers, the present work showed that pure poly-C aptamers may not have the highest affinity to hemagglutinin; introduction of stem and loop structure to the poly-C oligonucleotide showed better binding, as can be seen in Set-D; in addition, long poly-C (55 mer) had lower affinity than shorter poly-C (30 mer); both structural and thermodynamic consideration may cause these differing effects. Furthermore, it is clear that the anti-viral effect demonstrated in this study could not have been related only to the poly-anionic nature of oligonucleotides, since repeated G, CTC or CAC sequences, that share the same anionic nature but included G, T or A nucleotides, did not show high affinity to hemagglutinin.

The binding of DNA aptamers to influenza virus can be improved by introducing single stranded 2D loop elements enriched with C nucleotides. The scrambled (random) aptamers had similar binding affinity as the parent aptamer BV02 because they did not include single stranded loop elements enriched with C nucleotides. The affinity of the scrambled aptamers S1, S2 and S3 to the virus was governed by the relatively large size of 68 nucleotides, and for this reason BV02 showed its affinity.

The antiviral effect of our aptamers was not limited to the single strain of influenza BV02 was originally developed for; rather, the aptamers were active against a variety of strains, including influenza type B, whose hemagglutinin has only 27% amino-acid similarity to type A’s hemagglutinin. BV02 aptamer was originally selected based on its high affinity to a peptide fragment isolated from the hemagglutinin protein of a specific influenza strain [12], but its anti-influenza activity is wider and apparently relate to length of the aptamer.

It may be concluded that influenza viruses interacts with non-sequence-specific flexible-structure elements on the aptamer; these structural elements, however, are not entirely random. This mechanism of interaction is relevant to variety of influenza strains and may be applicable to other viruses as well.

In the future, other descriptors may be incorporated for better accuracy, describing, for example, chemical modifications, other secondary structures or energetic parameters. It may be worthwhile to test the importance of aptamer flexibility by incorporating abasic nucleotides (deoxyphosphribose without nucleobase) that provide maximal flexibility to the oligonucleotide, as well as other non-nucleotide synthetic oligomers.

This work lays the basis for a new structure-activity approach to aptamer design that may serve in the expending oligonucleic world. The work also reveals a special aptamer binding characteristic of the influenza virus hemagglutinin that may be targeted by new anti-viral agents, oligonucleotides or other non-small molecule oligomers.

## Supporting Information

Figure S1 **The inhibitory effect of aptamers BV35r and BV42 on influenza type B infection of MDCK cells.** Aptamers BV35r and BV42 inhibited the infection of influenza type B (B/PERTH/211/2001) with an EC50 of 5 and 30 nM, respectively. The amount of the virus attached to the cell culture is expressed as optic density (OD) of the colorimetric product that is produced by enzyme attached to anti-influenza antibody. The error bar indicated standard error (SE).
(TIF)

Figure S2 **The anti-viral effect of BV42 aptamer in different concentrations in influenza mouse model.** The anti-viral effect of aptamer BV42 at concentration 2–25 mg/Kg is presented. The severity of the infection was monitored according to the body weight change of each mouse along the experiment. Single intranasal treatment BV42 were administrated, PBS buffer served as placebo. The viral inoculum and treatment were co-administrated intranasally. The most effective treatment of BV42 was 10 mg/Kg. The statistics is based on ANOVA for repeated measures and Post-hoc Bonferroni’s statistical analysis, (**p<0.01, ***p<0.001). The error bar indicated standard error (SE). On day 4 the viral count reduction (based on qPCR) in mice lungs was about 4% with 2.5 & 5 mg/Kg treatments, 74% reduction with 10 mg/Kg treatment and 60% reduction with 25 mg/Kg treatment.
(TIF)

Table S1 **Aptamer list.**
(DOCX)

Table S2 **Descriptors list.**
(DOCX)

Data S1 (XLSX)

## References

[1] Hurt AC, Holien JK, Barr IG (2009) In vitro generation of neuraminidase inhibitor resistance in A(H5N1) influenza viruses. Antimicrob Agents Chemother 53: 4433–4440. 19651908 10.1128/AAC.00334-09 PMC2764219

[2] Ilyushina NA, Hoffmann E, Salomon R, Webster RG, Govorkova EA (2007) Amantadine-oseltamivir combination therapy for H5N1 influenza virus infection in mice. Antivir Ther 12: 363–370. 17591026

[3] Preziosi P (2011) Influenza pharmacotherapy: present situation, strategies and hopes. Expert Opin Pharmacother 12: 1523–1549. 21438743 10.1517/14656566.2011.566557

[4] Sherman KE, Flamm SL, Afdhal NH, Nelson DR, Sulkowski MS, et al (2011) Response-guided telaprevir combination treatment for hepatitis C virus infection. N Engl J Med 365: 1014–1024. 21916639 10.1056/NEJMoa1014463 PMC3809077

[5] Taubenberger JK, Morens DM (2010) Influenza: the once and future pandemic. Public Health Rep 125 Suppl 3 16–26. PMC2862331 20568566

[6] Weinstock DM, Zuccotti G (2009) The evolution of influenza resistance and treatment. JAMA 301: 1066–1069. 19255112 10.1001/jama.2009.324

[7] Ekiert DC, Bhabha G, Elsliger MA, Friesen RH, Jongeneelen M, et al (2009) Antibody recognition of a highly conserved influenza virus epitope. Science 324: 246–251. 19251591 10.1126/science.1171491 PMC2758658

[8] Hayden FG (2013) Newer influenza antivirals, biotherapeutics and combinations. Influenza Other Respi Viruses 7 Suppl 1 63–75. 10.1111/irv.12045 PMC5978626 23279899

[9] Sui J, Hwang WC, Perez S, Wei G, Aird D, et al (2009) Structural and functional bases for broad-spectrum neutralization of avian and human influenza A viruses. Nat Struct Mol Biol 16: 265–273. 19234466 10.1038/nsmb.1566 PMC2692245

[10] Triana-Baltzer GB, Gubareva LV, Nicholls JM, Pearce MB, Mishin VP, et al (2009) Novel pandemic influenza A(H1N1) viruses are potently inhibited by DAS181, a sialidase fusion protein. PLoS One 4: e7788. 19893747 10.1371/journal.pone.0007788 PMC2770640

[11] Belser JA, Lu X, Szretter KJ, Jin X, Aschenbrenner LM, et al (2007) DAS181, a novel sialidase fusion protein, protects mice from lethal avian influenza H5N1 virus infection. J Infect Dis 196: 1493–1499. 18008229 10.1086/522609

[12] Jeon SH, Kayhan B, Ben-Yedidia T, Arnon R (2004) A DNA aptamer prevents influenza infection by blocking the receptor binding region of the viral hemagglutinin. J Biol Chem 279: 48410–48419. 15358767 10.1074/jbc.M409059200

[13] Bouchard PR, Hutabarat RM, Thompson KM (2010) Discovery and development of therapeutic aptamers. Annu Rev Pharmacol Toxicol 50: 237–257. 20055704 10.1146/annurev.pharmtox.010909.105547

[14] Stoltenburg R, Reinemann C, Strehlitz B (2007) SELEX–a (r)evolutionary method to generate high-affinity nucleic acid ligands. Biomol Eng 24: 381–403. 17627883 10.1016/j.bioeng.2007.06.001

[15] Keefe AD, Pai S, Ellington A (2010) Aptamers as therapeutics. Nat Rev Drug Discov 9: 537–550. 20592747 10.1038/nrd3141 PMC7097324

[16] Binning JM, Leung DW, Amarasinghe GK (2012) Aptamers in virology: recent advances and challenges. Front Microbiol 3: 29. 22347221 10.3389/fmicb.2012.00029 PMC3274758

[17] Aquino-Jarquin G, Toscano-Garibay JD (2012) RNA aptamer evolution: two decades of SELEction. Int J Mol Sci 12: 9155–9171. 10.3390/ijms12129155 PMC3257122 22272125

[18] Huynen MA (1996) Exploring phenotype space through neutral evolution. J Mol Evol 43: 165–169. 8703081 10.1007/BF02338823

[19] Bing T, Yang X, Mei H, Cao Z, Shangguan D (2010) Conservative secondary structure motif of streptavidin-binding aptamers generated by different laboratories. Bioorg Med Chem 18: 1798–1805. 20153201 10.1016/j.bmc.2010.01.054

[20] Fischer NO, Tok JB, Tarasow TM (2008) Massively parallel interrogation of aptamer sequence, structure and function. PLoS One 3: e2720. 18628955 10.1371/journal.pone.0002720 PMC2444025

[21] Platt M, Rowe W, Knowles J, Day PJ, Kell DB (2009) Analysis of aptamer sequence activity relationships. Integr Biol (Camb) 1: 116–122. 20023798 10.1039/b814892a

[22] Roulet E, Busso S, Camargo AA, Simpson AJ, Mermod N, et al (2002) High-throughput SELEX SAGE method for quantitative modeling of transcription-factor binding sites. Nat Biotechnol 20: 831–835. 12101405 10.1038/nbt718

[23] Rockey WM, Hernandez FJ, Huang SY, Cao S, Howell CA, et al (2011) Rational truncation of an RNA aptamer to prostate-specific membrane antigen using computational structural modeling. Nucleic Acid Ther 21: 299–314. 22004414 10.1089/nat.2011.0313 PMC3198747

[24] Delisi C, Crothers DM (1971) Prediction of RNA secondary structure. Proc Natl Acad Sci U S A 68: 2682–2685. 5288243 10.1073/pnas.68.11.2682 PMC389500

[25] Nussinov R, Pieczenik G, Griggs JR, Kleitman DJ (1978) Algorithms for Loop Matchings. SIAM J Appl Math 35: 68–82.

[26] Zuker M (2003) Mfold web server for nucleic acid folding and hybridization prediction. Nucleic Acids Res 31: 3406–3415. 12824337 10.1093/nar/gkg595 PMC169194

[27] Mathews DH, Disney MD, Childs JL, Schroeder SJ, Zuker M, et al (2004) Incorporating chemical modification constraints into a dynamic programming algorithm for prediction of RNA secondary structure. Proc Natl Acad Sci U S A 101: 7287–7292. 15123812 10.1073/pnas.0401799101 PMC409911

[28] Zuker M, Stiegler P (1981) Optimal computer folding of large RNA sequences using thermodynamics and auxiliary information. Nucleic Acids Res 9: 133–148. 6163133 10.1093/nar/9.1.133 PMC326673

[29] Hofacker IL, Fontana W, Stadler PF, Bonhoeffer LS, Tacker M, et al (1994) Fast folding and comparison of RNA secondary structures. MonatshChem 125: 167–188.

[30] Sidwell RW, Smee DF (2000) In vitro and in vivo assay systems for study of influenza virus inhibitors. Antiviral Res 48: 1–16. 11080536 10.1016/s0166-3542(00)00125-x

[31] SantaLucia J Jr (1998) A unified view of polymer, dumbbell, and oligonucleotide DNA nearest-neighbor thermodynamics. Proc Natl Acad Sci U S A 95: 1460–1465. 9465037 10.1073/pnas.95.4.1460 PMC19045

[32] Peyret N (2000) Prediction of Nucleic Acid Hybridization: Parameters and Algorithms. Detroit, MI.: Wayne State University.

[33] Golbraikh A, Shen M, Xiao Z, Xiao YD, Lee KH, et al (2003) Rational selection of training and test sets for the development of validated QSAR models. J Comput Aided Mol Des 17: 241–253. 13677490 10.1023/a:1025386326946

[34] Kapetanovic IM (2008) Computer-aided drug discovery and development (CADDD): in silico-chemico-biological approach. Chem Biol Interact 171: 165–176. 17229415 10.1016/j.cbi.2006.12.006 PMC2253724

[35] Choi SK, Lee C, Lee KS, Choe SY, Mo IP, et al (2012) DNA aptamers against the receptor binding region of hemagglutinin prevent avian influenza viral infection. Mol Cells 32: 527–533. 10.1007/s10059-011-0156-x PMC3887679 22058017

[36] Vaillant A, Juteau JM, Lu H, Liu S, Lackman-Smith C, et al (2006) Phosphorothioate oligonucleotides inhibit human immunodeficiency virus type 1 fusion by blocking gp41 core formation. Antimicrob Agents Chemother 50: 1393–1401. 16569857 10.1128/AAC.50.4.1393-1401.2006 PMC1426958

[37] Matsumura T, Hu Z, Kato T, Dreux M, Zhang YY, et al (2009) Amphipathic DNA polymers inhibit hepatitis C virus infection by blocking viral entry. Gastroenterology 137: 673–681. 19394333 10.1053/j.gastro.2009.04.048 PMC2803092

[38] Bernstein DI, Goyette N, Cardin R, Kern ER, Boivin G, et al (2008) Amphipathic DNA polymers exhibit antiherpetic activity in vitro and in vivo. Antimicrob Agents Chemother 52: 2727–2733. 18505857 10.1128/AAC.00279-08 PMC2493138

[39] Cardin RD, Bravo FJ, Sewell AP, Cummins J, Flamand L, et al (2009) Amphipathic DNA polymers exhibit antiviral activity against systemic murine Cytomegalovirus infection. Virol J 6: 214. 19954538 10.1186/1743-422X-6-214 PMC2794273

[40] Baba M, Pauwels R, Balzarini J, Arnout J, Desmyter J, et al (1988) Mechanism of inhibitory effect of dextran sulfate and heparin on replication of human immunodeficiency virus in vitro. Proc Natl Acad Sci U S A 85: 6132–6136. 2457906 10.1073/pnas.85.16.6132 PMC281919

[41] De Clercq E (2001) 2001 ASPET Otto Krayer Award Lecture. Molecular targets for antiviral agents. J Pharmacol Exp Ther 297: 1–10. 11259521

[42] Este JA, Schols D, De Vreese K, Van Laethem K, Vandamme AM, et al (1997) Development of resistance of human immunodeficiency virus type 1 to dextran sulfate associated with the emergence of specific mutations in the envelope gp120 glycoprotein. Mol Pharmacol 52: 98–104. 9224818 10.1124/mol.52.1.98

[43] Neurath AR, Strick N, Li YY (2002) Anti-HIV-1 activity of anionic polymers: a comparative study of candidate microbicides. BMC Infect Dis 2: 27. 12445331 10.1186/1471-2334-2-27 PMC139971

[44] Gopinath SC, Misono TS, Kawasaki K, Mizuno T, Imai M, et al (2006) An RNA aptamer that distinguishes between closely related human influenza viruses and inhibits haemagglutinin-mediated membrane fusion. J Gen Virol 87: 479–487. 16476969 10.1099/vir.0.81508-0

[45] Park SY, Kim S, Yoon H, Kim KB, Kalme SS, et al (2011) Selection of an antiviral RNA aptamer against hemagglutinin of the subtype H5 avian influenza virus. Nucleic Acid Ther 21: 395–402. 22017542 10.1089/nat.2011.0321

