# Supplementary figures and images for: Designing Anti-Influenza Aptamers: Novel Quantitative Structure Activity Relationship Approach Gives Insights into Aptamer – Virus Interaction
Source: PLoS One. 2014 May 20;9(5):e97696. doi: 10.1371/journal.pone.0097696 (PMC4028238; doi:10.1371/journal.pone.0097696)

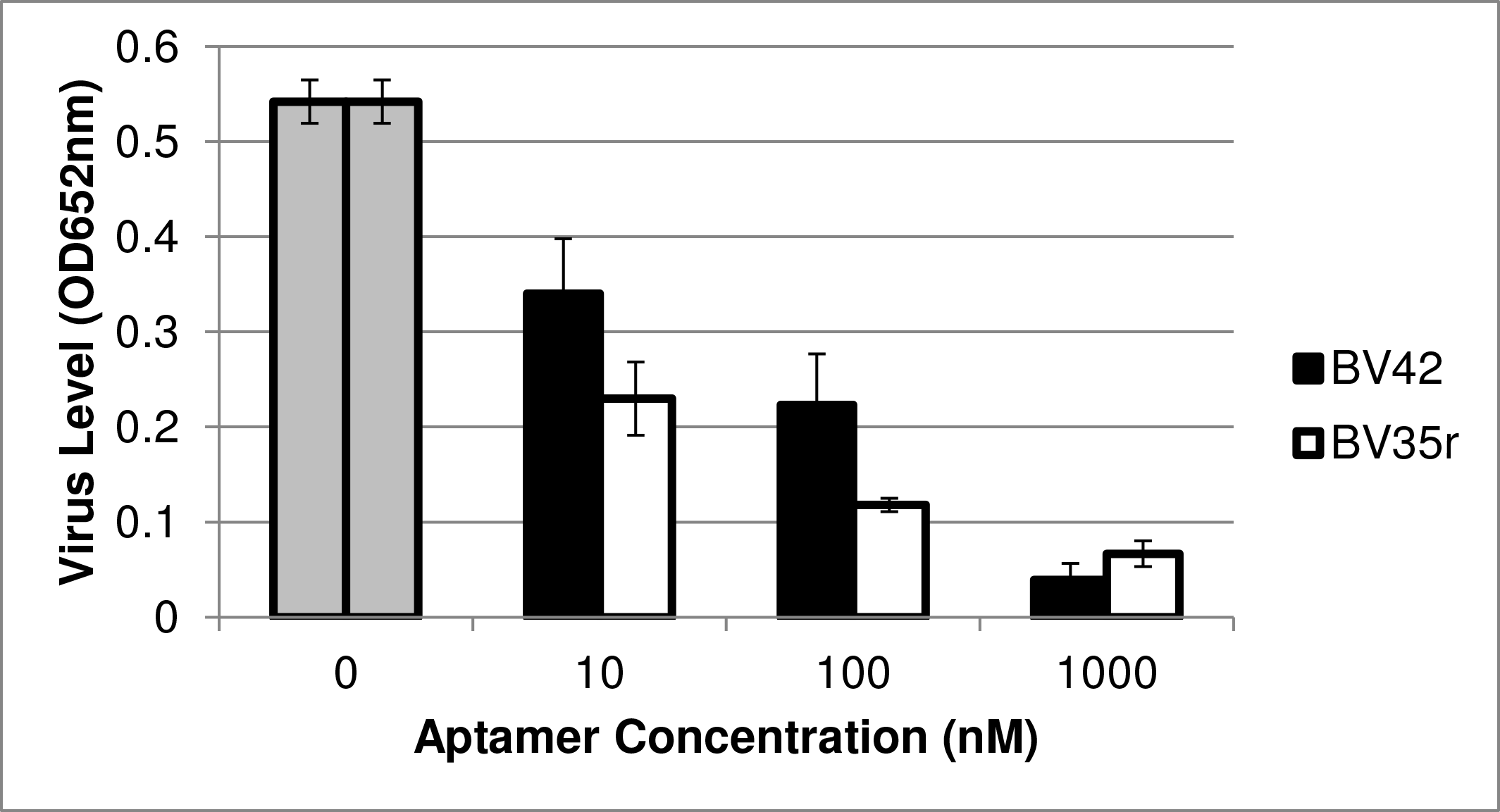

Supplement: Figure S1 — The inhibitory effect of aptamers BV35r and BV42 on influenza type B infection of MDCK cells. Aptamers BV35r and BV42 inhibited the infection of influenza type B (B/PERTH/211/2001) with an EC50 of 5 and 30 nM, respectively. The amount of the virus attached to the cell culture is expressed as optic density (OD) of the colorimetric product that is produced by enzyme attached to anti-influenza antibody. The error bar indicated standard error (SE). (TIF) [file pone.0097696.s001.tif]

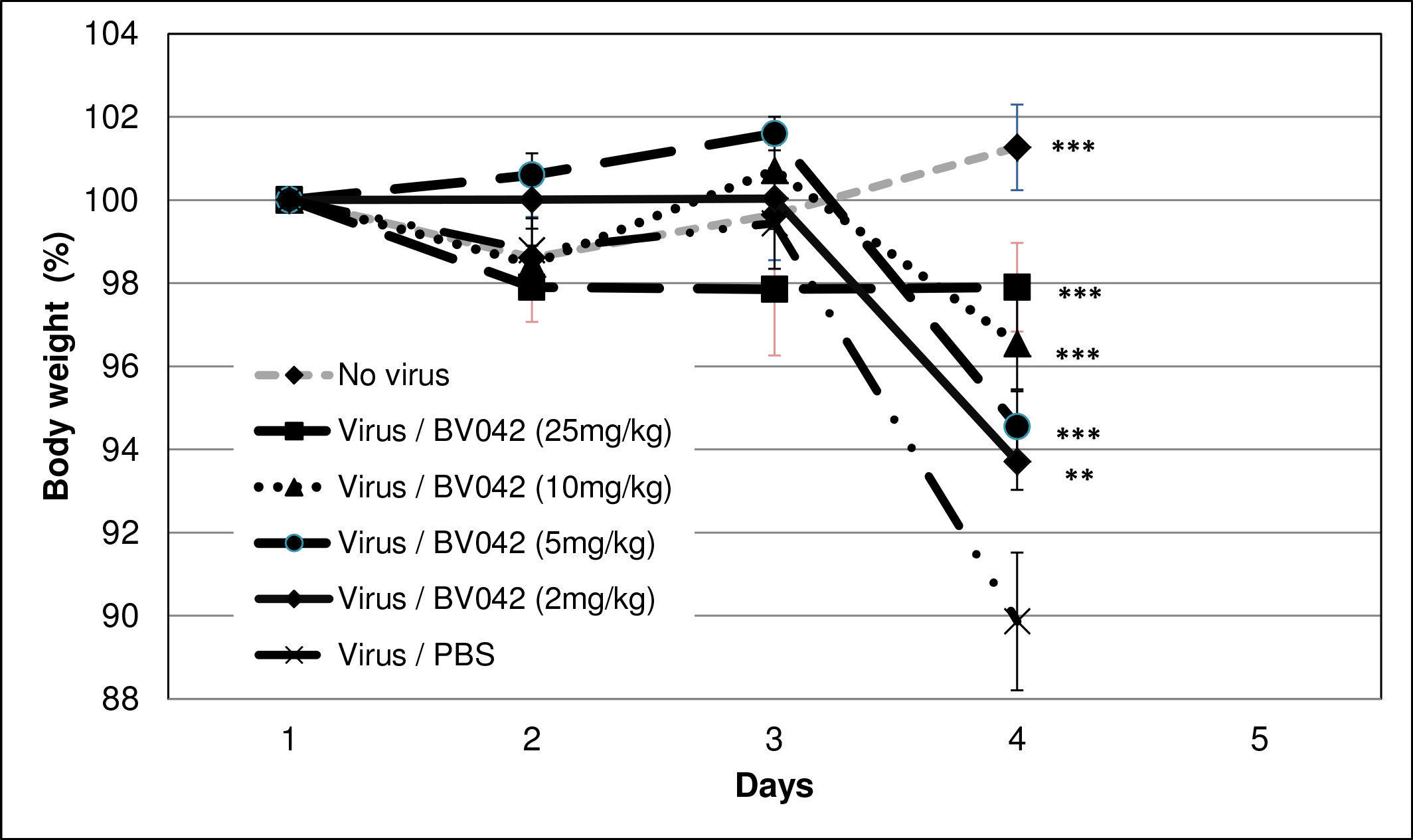

Supplement: Figure S2 — The anti-viral effect of BV42 aptamer in different concentrations in influenza mouse model. The anti-viral effect of aptamer BV42 at concentration 2–25 mg/Kg is presented. The severity of the infection was monitored according to the body weight change of each mouse along the experiment. Single intranasal treatment BV42 were administrated, PBS buffer served as placebo. The viral inoculum and treatment were co-administrated intranasally. The most effective treatment of BV42 was 10 mg/Kg. The statistics is based on ANOVA for repeated measures and Post-hoc Bonferroni’s statistical analysis, (**p<0.01, ***p<0.001). The error bar indicated standard error (SE). On day 4 the viral count reduction (based on qPCR) in mice lungs was about 4% with 2.5 & 5 mg/Kg treatments, 74% reduction with 10 mg/Kg treatment and 60% reduction with 25 mg/Kg treatment. (TIF) [file pone.0097696.s002.tif]
