# Supplementary material for: Designing Anti-Influenza Aptamers: Novel Quantitative Structure Activity Relationship Approach Gives Insights into Aptamer – Virus Interaction
Source: PLoS One. 2014 May 20;9(5):e97696. doi: 10.1371/journal.pone.0097696 (PMC4028238; doi:10.1371/journal.pone.0097696)
Supplement: Table S1 — Aptamer list. (DOCX) [file pone.0097696.s003.docx]

Table 1 supplement

**Aptamer list**

| Name | Set  (1=training 2=test) | Binding IC50 A22/Aptamer | Sequence 5’ 🡪 3’ ssDNA |
| --- | --- | --- | --- |
| BV02 | 1 | 1 | AATTAACCCTCACTAAAGGGCTGAGTCTCAAAACCGCAATACACTGGTTGTATGGTCGAATAAGTTAA |
| BV02a | 2 | 0.946 | AATTAACCCTCACTAAAGGGCTGATGCGGAATAAGTTAAAACCGCAGGTATACACTGGTTGTATCGAA |
| BV11 | 1 | 0.124 | AATTAACCCTCACTAAAGGGCTGAGTCTCAAAACGTTTTGAGACTCAGCCCTTTAGTGAGGGTTAATT |
| BV13 | 1 | 0.084 | GTTTTGAGACTCAGCCCGGGCTGAGTCTCAAAACCGCAATACACTGGTTGTACAACCAGTGTATTGCG |
| BV14 | 2 | 1.333 | AATTAACCCTCACTAAAGGGCTGAGTCTCAAAACCGCAATACACTGGTTGTACAACCAGTGTATTGCG |
| BV18 | 1 | 7.258 | AATTAACCCTCACTAAAGGGCTGAGTCTCAAAACCGCAATACACTGGTTGTATGTTAATTGGGAGTGA |
| BV19 | 1 | 1.345 | AATTAACCCTCACTAAAGGGCTGAGTCTCAAAACCGCAATACACTCAGCCCTTTAGTGAGGGTTAATT |
| BV19a | 1 | 0.574 | AATTAACCCTCACTAAAGGGCTCTCACTCAAAACCGCAATACACTCAGCCCTTTAGTGAGGGTTAATT |
| BV19b | 1 | 1.263 | AATTAACCCTCACTAAAGCCGACTCACTCAAAACCGCAATACACTCAGCCCTTTAGTGAGGGTTAATT |
| BV19c | 1 | 0.086 | GGCTCACTAAAGGGCTGAGTCTCAAAACCGCAATACACTCAGCCCTTTAGTGAGGGTTAATTTTAAAGG |
| BV19f | 1 | 0.131 | AACCCTCACTCCGACTCACTCAAAACCGCAATACACTCAGCCAGTGAGGGTT |
| BV19g | 2 | 0.132 | CTCACTAAAGGGCTGAGTCTCAAAACCGCAATACACTCAGCCCTTTAGTGAG |
| BV19h | 2 | 0.016 | AACCCTCACTCTCAAAACCGCAATACAGTGAGGGTT |
| BV19i | 2 | 0.071 | AATTAACCCTGACTAAAGGGGTGAGTCTCAAAACCGCAATACACTCAGCCCTTTAGTGAGGGTTAATT |
| BV19j | 1 | 0.071 | AATTAAGCCTGACTTAAGGGGTGAGTCTCAAAACCGCAATACACTCAGCCCTTTAGTGAGGGTTAATT |
| BV19k | 1 | 0.932 | AAATAAGCCTGACTTAACGGGTGACTCTCAAAACCGCAATACACTCAGCCCTTTAGTGAGGGTTAATT |
| BV19l | 1 | 0.004 | AATTAACCCTCCTAAAGGGTCTGAGTCTCAAAACCGCAATACACTCAGCCCTTTAGTGAGGGTTAATT |
| BV24 | 1 | 15.077 | CCCCCCCCCCCCCCCCCCCCCCCCCCCCCC |
| BV24a | 1 | 0.080 | CTCCTCCTCCTCCTCCTCCTCCTCCTCCTC |
| BV24b | 1 | 0.050 | CACCACCACCACCACCACCACCACCACCAC |
| BV24c | 1 | 4.873 | CCCCCCCCCCCCCCCCCCCCCCCCCCCCCCCCCCCCCCCCCCCCCCCCCCCCCCC |
| BV24d | 1 | 0.106 | GGGGGGGGGGGGGGGGGGGGGGGGGGGGGGGGGGGGGGGGGGGGGGGGGGGGGGG |
| BV24e | 1 | 11.573 | GGGGGGCCCCCCCCCCCCCCCCCCCCCCCCCCCCCCCCCCCCCCCCCCCCCCCCC |
| BV24f | 1 | 6.731 | C*C*C*CCCCCC*CCCCCC*CCCCCC*CCCCCC*CCCCCC*CCCCCC*CCCCCC*CCCCCCC*C*C*C (*= phosphothioate ) |
| BV26 | 1 | 0.792 | GCTGAGTCTCAAAACCGCAATACACTGGTT |
| BV27 | 2 | 0.116 | CTCACTAAAGGGCTGAGTCAAACCGCAATACACTGGTTGTATGGTCGAATAAGTT |
| BV27c | 1 | 0.771 | CTCACTAAAGGGCTGAGTCAAACCGCAATACACTGGTTGTATGGTCGGTTAAGTT |
| BV27f | 2 | 0.750 | GTCACTAAAGGGCTGAGTCAAACCGCAATACACTGGTTGTATGGTCGGTTAAGTT |
| BV27g | 1 | 0.093 | TCCTCAGTAAAGGGCTGAGAAACCGCAATACACTGGTAAAAAGTGCGGTTAAGTT |
| BV27h | 1 | 0.092 | TCCTCAGTAAAGGGCTGAGTCAAACCGCAATACACTGGTAAAAAGTGCGGTTAAG |
| BV27i | 1 | 0.065 | TCCTCAGTAAAGGGCTGAGTCAAAACCGCAATACACTGGTAAAAAGTGCGGTTAG |
| BV27j | 2 | 0.089 | GTCACTAAAGGGCTGAGTCAAACCGCCATACACTGGTTGTATGGCGGTTAAGTT |
| BV27m | 1 | 0.012 | GGCTGAGTCAAACCGCCTAACACTGGTACAATGGCGGTTAAGTT |
| BV27n | 1 | 0.151 | TCCTCAGTAAAATAGCGGGGCTGAGTCAAACCGCAATACACTGGTAAAAAGTGCGGTTAAG |
| BV27o | 1 | 0.116 | TCATTCTCAGTAAAGGGCTGAGTCAAACCGCAATACACTGGTAAAAAGTGCGGTTAGCGAG |
| BV27p | 1 | 0.133 | TCATTCTCAGTAAAGGGCTGAGAATTCAAACCGCAATACACTGGTAAAAAGTGCGGTTAAG |
| BV28 | 1 | 1.358 | A*T*T*AACCCTCACTAAAGGGCTGAGTCTCAAAACCGCAATACACTGGTTGTATGGTCGAATAAGT*T*A*A |
| BV29 | 1 | 6.704 | A*T*T*AACCCTCACTAAAGGGCTGAGTCTCAAAACCGCAATACACTGGTTGTATGGTCGAATAAGTTAA-3InvdT |
| BV31 | 1 | 7.512 | A*T*T*AACC*CTCACTAAAGGG*CTGAGTC*TCAAAACCG*CAATACACTG*GTTGTATG*GTCGAATAAGT*T*A*A |
| BV32 | 1 | 1.334 | [PEG200]ATTAACCCTCACTAAAGGGCTGAGTCTCAAAACCGCAATACACTGGTTGTATGGTCGAATAAGTTAA |
| BV35 | 1 | 1.011 | CTCACTAAAGCGCTGAGTCCCCCCCCCCCCCCCCCCCACTCAGCGCTTTAGTGAG |
| BV35a | 1 | 14.570 | AACGCTCACTCCCCCCCCCCCCCCCCCCCCCCCCCCCCCCCCCCCAGTGAGCGTT |
| BV35b | 1 | 0.938 | CCCCCCCCCGCGCTGAGTCCCCCCCCCCCCCCCCCCCACTCAGCGCGCCCCCCCC |
| BV35c | 1 | 1.159 | CCCCCCCCCCCCCCCCCGCGCTGAGTCCCCCCCCCCCCCCCCCCCACTCAGCGCG |
| BV35d | 1 | 0.999 | AACGCTCACTCCCCCCCCCCCCCCCCCCCCCCCCCCCCCCCAGTGAGCGTT |
| BV35e | 1 | 0.942 | AACGCTCACTCCCCCCCCCCCCCCCCCCCCCCCCCCCAGTGAGCGTT |
| BV35f | 2 | 3.318 | AACGCTCACTCCCCCCCCCCCCCCCCCCCCCCCAGTGAGCGTT |
| BV35g | 1 | 0.639 | AACGCTCACTCCCCCCCCCCCCCCCCCCCAGTGAGCGTT |
| BV35h | 2 | 10.406 | AACGCTCACTCCCCCCCCCCCCCCCCCCCCCCCCCCCCCCCCCCCCCCCAGTGAGCGTT |
| BV35i | 2 | 6.063 | ACGCTCACTCCCCCCCCCCCCCCCCCCCCCCCCCCCCCCCCCCCAGTGAGCGT |
| BV35j | 1 | 4.382 | CGCTCACTCCCCCCCCCCCCCCCCCCCCCCCCCCCCCCCCCCCAGTGAGCG |
| BV35k | 2 | 17.547 | CGCCACTCCCCCCCCCCCCCCCCCCCCCCCCCCCCCCCCCCCAGTGGCG |
| BV35l | 1 | 5.441 | ACACGCTCACTCCCCCCCCCCCCCCCCCCCCCCCCCCCCCCCCCCCAGTGAGCGTGT |
| BV35m | 1 | 6.545 | AACGCTCACTCCCCCACCCCCCCACCCCCCCACCCCCCCACCCCCAGTGAGCGTT |
| BV35n | 2 | 7.234 | AACGCTCACTCCCCCCCCACCCCCCCCACCCCCCCCACCCCCCCCAGTGAGCGTT |
| BV35o | 1 | 9.917 | AACGCTCACTCCCCCCCCCCCACCCCCCCCCCCACCCCCCCCCCCAGTGAGCGTT |
| BV35p | 1 | 9.884 | AACGCTCACTCCCCCCCCCCCCCCCCCACCCCCCCCCCCCCCCCCAGTGAGCGTT |
| BV35q | 1 | 14.126 | AACGCTCACTCCCCCCCACCCCCCCCCCCCCCCCCCCCCCCCCCCAGTGAGCGTT |
| BV35r | 1 | 11.331 | AACGCTCACTCCCCCAAGAACCCCCCCCCCCCCCCCCCCCCCCCCAGTGAGCGTT |
| BV35s | 1 | 7.454 | AACGCTCACTCCCCCCCCCCCCCCCAAGAACCCCCCCCCCCCCCCAGTGAGCGTT |
| BV36a | 1 | 0.780 | AATTAACGCTCACTAAAGCCCCCCCCCCCCCCCCCCCCCCCCCCCCCCCCCTTTAGTGAGCGTTAATT |
| BV36b | 1 | 0.611 | AATTAACGCTCACTAAAGCCCCACCCACCCACCCACCCACCCACCCACCCCTTTAGTGAGCGTTAATT |
| BV36c | 2 | 14.362 | CCCCAACGCTCACTAAAGCCCCCCCCCCCCCCCCCCCCCCCCCCCCCCCCCTTTAGTGAGCGTTCCCC |
| BV37a | 2 | 0.874 | TTCCTCAGCAAAATAGCGGGGCTGAGCAACCGCAATACACTGGTAAAAAGTGCGGTTAAGA |
| BV37b | 2 | 1.000 | TCCTCAGCAAAATAGCGGGGCTGAGTCAAACCGCAATACACTGGTAAAAAGTGCGGTTAAG |
| BV37c | 2 | 7.560 | TCCTCAGCAAAATAGCGGGGCTGAGTCTCAAACCGCAATACACTGGTAAAAAGTGCGGTTA |
| BV37e | 1 | 0.995 | TCTCAGCCCCCGCTGAGTCAAAGCGCACCCCCCCCCCCCCCCCTGCGCTTAA |
| BV37f | 1 | 0.495 | TCTCAGCCCCCGCTGAGTCAAAGCGCACCCCCCCCCCCCCCCCCCCTGCGCTTAA |
| BV40 | 2 | 21.217 | CGCCACTCCCCCCCCAAGAACCCCCCCCCCCCCCCCCCCCCCAGTGGCG |
| BV40a | 2 | 24.750 | CGCCACTCCCCCCCAAGAACCCCCCCCCCCCCCCCCCCCCCCAGTGGCG |
| BV40c | 1 | 23.313 | CGCCACTCCCCCAAGAACCCCCCCCCCCCCCCCCCCCCCCCCAGTGGCG |
| BV40d | 1 | 23.489 | CGCCACTCCCCAAGAACCCCCCCCCCCCCCCCCCCCCCCCCCAGTGGCG |
| BV40e | 1 | 19.472 | CGCCACTCCCCCAAGAAGAACCCCCCCCCCCCCCCCCCCCCCCCCAGTGGCG |
| BV41 | 1 | 16.304 | AACGCCACTCCCCCCCCCCCCCCCCCCCCCCCCCCCCCCCCCCCAGTGGCGTT |
| BV41a | 1 | 18.929 | AACGCCACTCCCCCCAAGAACCCCCCCCCCCCCCCCCCCCCCCCAGTGGCGTT |
| BV42 | 1 | 19.648 | AACGCTCACTCCCCCAAGAAGAACCCCCCCCCCCCCCCCCCCCCCAGTGAGCGTT |
| BV42a | 1 | 15.909 | AACGCTCACTCCCCAAGAAGAAGAACCCCCCCCCCCCCCCCCCCCAGTGAGCGTT |
| BV42b | 1 | 14.926 | AACGCTCACTCCCCCCCCCCCCCCAAGAAGAACCCCCCCCCCCCCAGTGAGCGTT |
| BV43a | 1 | 11.564 | AACGCTCACTCCCAGAAGAAGAAGACCCCCCCCCCCCCCCCCCCCAGTGAGCGTT |
| BV43b | 1 | 8.011 | AACGCTCACTCCCAAGAAGAAGAACCCCCCCCCCCCCCCCAGTGAGCGTT |
| BV44 | 1 | 25.639 | AACGCTCACTCCCCCCCCCCCCCCCCCCCCCCCCCCCCCCCCCCCAACGCTCACT |
| BV44a | 1 | 21.761 | AGTGAGCGTTCCCCCCCCCCCCCCCCCCCCCCCCCCCCCCCCCCCAGTGAGCGTT |
| BV44b | 1 | 12.691 | CCCCCAAGAACCCCCCCCCCCCCCCCCCCCCCCCCAGTGAGCGTT |
| BV45 | 1 | 14.188 | AACGCTCACTAACCCCCCCCCCACCCCCCCCCCACCCCCCCCCCAAGTGAGCGTT |
| BV45a | 1 | 12.553 | AACGCTCACTAAACCCCCCCCCACCCCCCCCCACCCCCCCCCAAAAGTGAGCGTT |
| BV45b | 2 | 15.081 | AACGCTCACTACCCCCCCCCCAACCCCCCCCCCAACCCCCCCCCCAGTGAGCGTT |
| BV45c | 2 | 11.672 | AACGCTCACTCCCCCAAGAACCCCCCCCCCCCTCCCCCCCCCCCCAGTGAGCGTT |
| BV45d | 1 | 10.654 | AACGCTCACTCCCCCAAGAACCCCCCCCCCCTGACCCCCCCCCCCAGTGAGCGTT |
| BV45e | 2 | 11.446 | AACGCTCACTCCCCCAAGAACCCCCCCCCCATGCACCCCCCCCCCAGTGAGCGTT |
| BV46 | 1 | 12.943 | AACGCTCACTCCCCCAAGAACCCCCCCCCCCCCCCCCCCCCCCCAGTGAGCGTT |
| BV46a | 2 | 12.792 | AACGCTCACTCCCCCAAGAACCCCCCCCCCCCCCCCCCCCCCCAGTGAGCGTT |
| BV46b | 1 | 12.213 | AACGCTCACTCCCCCAAGAACCCCCCCCCCCCCCCCCCCCCCAGTGAGCGTT |
| BV46c | 1 | 10.616 | AACGCTCACTCCCCCAAGAACCCCCCCCCCCCCCCCCCCCCAGTGAGCGTT |
| BV46d | 1 | 11.522 | AACGCTCACTCCCCCAAGAACCCCCCCCCCCCCCCCCCCCCCCCAGTGAGCGTT |
| S1 | Non | 1.1 | GAAATAACCTCTGATAAAAAATTTCTCCATAGCGAAGTAAGTCCATAAGGAATGCCGATTCGGCTTGC |
| S2 | Non | 1.0 | AGATAAATGTCCGGGTTCGCTTCACTGATCAAACATAGACAGGTATAATTACTGCCTGAAGAAAATCC |
| S3 | Non | 0.7 | GTAAATACCTCTGAAATAATATCTCCATGACAGAGTAAGTCCATGAGAACTGACGATGCAAGTCTGTC |
| C7-35M | 2 | 0.549 | GGTAGTTATAGTATATGGAAGGGGGTGTCGTATGG |
| C7C | 1 | 15.547 | GGTAGTTATAGTATATGGAAGGGGCCCCCCCCCCCCCCCCGTGTCGTATGG |
|  |  |  |  |
