# Supplementary material for: Designing Anti-Influenza Aptamers: Novel Quantitative Structure Activity Relationship Approach Gives Insights into Aptamer – Virus Interaction
Source: PLoS One. 2014 May 20;9(5):e97696. doi: 10.1371/journal.pone.0097696 (PMC4028238; doi:10.1371/journal.pone.0097696)
Supplement: Table S2 — Descriptors list. (DOCX) [file pone.0097696.s004.docx]

Table 2 Supplement

**Descriptors list**

The descriptors that were extracted from the calculated structures included:

| Name | Descriptor explanation |
| --- | --- |
|  | **General Descriptors (1D)** |
| x1 | Total number of Bases |
| x2 | Number of calculated conformations |
| x3 | Number of Chemical modification |
| x4 | Total number of A nucleotides |
| x5 | Total number of T nucleotides |
| x6 | Total number of C nucleotides |
| x7 | Total number of G nucleotides |
| x8 | Number of Pohsphothioated modification |
| x9 | Existence of PEG modification |
| x10 | Existence of 3InvdT modification |
|  | **Thermodynamic descriptors** |
| x11 | Free Energy δG calculated |
| x12 | Enthalpy δH calculated |
| x13 | Entropy δS calculated |
| x14 | Tm calculated melting point |
|  | **C-Stretches related descriptors** |
| x15 | Length of longest un-paired C-stretch that may be perturbed with any sequence that is no longer than 8 nucleotides |
| x16 | Number of nucleotides that participate in perturbations of the C-stretches |
| x17 | Sum of un-paired C-stretches located in external loops |
| x18 | Length of longest un-paired C-stretches located in loops |
| x19 | Length of longest un-paired C-stretches |
| x20 | Sum of un-paired C-stretches comprised of 3 Cs or more |
| x21 | Sum of un-paired C-stretches comprised of 3 Cs or more located in loops |
| x22 | The proportion of Cs in the largest loop |
| x23 | orientation of disturbance in largest loop (1=180deg; 2=125 3=90 4=45 6= scattered or non) |
| x24 | Position of the C-stretch (S=stem L=loop E=external loop) |
|  | **Un-paired nucleotides descriptors** |
| x25 | Sum of un-paired nucleotides in external loops |
| x26 | Free energy contribution of external loop |
| x27 | Maximal length of external loops |
| x28 | Sum of all un-paired nucleotides |
| x29 | Number of stem & loops |
| x30 | Hinge: length of un-paired nucleotides separating the longest stems |
|  | **Largest loop descriptors** |
| x31 | Size of largest loop |
| x32 | δG of largest loop |
| x33 | bp closing pairs of largest loop |
| x34 | δG closing pairs of largest loop |
| x35 | closing pairs name of largest loop |
| x36 | number of distortion motives in stem of largest loop |
| x37 | number of un-paired basis making the distortion of the stem of largest loop |
| x38 | δG stem interior or Bulge loops |
|  | **Second largest loop descriptors** |
| x39 | Size of second largest loop |
| x40 | δG of second largest loop |
| x41 | bp closing pairs of second largest loop |
| x42 | δG closing pairs of second largest loop |
| x43 | closing pairs name of second largest loop |
| x44 | number of distortion motives in stem of second largest loop |
| x45 | number of un-paired basis making the distortion of the stem of second largest loop |
| x46 | δG stem interior or Bulge loops of second |
|  | **Third largest loop descriptors** |
| x47 | Size of third largest loop |
| x48 | δG of third largest loop |
| x49 | bp closing pairs of third largest loop |
| x50 | δG closing pairs of third largest loop |
| x51 | closing pairs name of third largest loop |
| x52 | number of distortion motives in stem of third largest loop |
|  | **Ratio descriptors (function of the above descriptors)** |
| f_1 | Ratio between Sum of all un-paired nucleotides And Total base number |
| f_2 | Ratio between Size of largest loop And Total base number |
| f_3 | Ratio between Size of largest loop And Size of second largest loop |
| f_4 | Ratio between total C nucleotides And Total base number |
| f_5 | Number of C nucleotides in largest loop |
| f_6 | Ratio between Length of longest un-paired C-stretch that may be perturbed with any sequence that is no longer than 8 nucleotides And Total base number |
| f_7 | Ratio between Sum of un-paired C-stretches comprised of 3 Cs or more located in loops And Total base number |
| f_8 | Ratio between Sum of un-paired C-stretches comprised of 3 Cs or more And Total base number |
